# Evaluation of multifarious plant growth promoting traits, antagonistic potential and phylogenetic affiliation of rhizobacteria associated with commercial tea plants grown in Darjeeling, India

**DOI:** 10.1371/journal.pone.0182302

**Published:** 2017-08-03

**Authors:** Jintu Dutta, Debajit Thakur

**Affiliations:** Microbial Biotechnology Laboratory, Life Sciences Division, Institute of Advanced Study in Science and Technology, Guwahati, Assam, India; Agroecological Institute, CHINA

## Abstract

Plant growth promoting rhizobacteria (PGPR) are studied in different agricultural crops but the interaction of PGPR of tea crop is not yet studied well. In the present study, the indigenous tea rhizobacteria were isolated from seven tea estates of Darjeeling located in West Bengal, India. A total of 150 rhizobacterial isolates were screened for antagonistic activity against six different fungal pathogens i.e. *Nigrospora sphaerica* (KJ767520), *Pestalotiopsis theae* (ITCC 6599), *Curvularia eragostidis* (ITCC 6429), *Glomerella cingulata* (MTCC 2033), *Rhizoctonia Solani* (MTCC 4633) and *Fusarium oxysporum* (MTCC 284), out of which 48 isolates were antagonist to at least one fungal pathogen used. These 48 isolates exhibited multifarious antifungal properties like the production of siderophore, chitinase, protease and cellulase and also plant growth promoting (PGP) traits like IAA production, phosphate solubilization, ammonia and ACC deaminase production. Amplified ribosomal DNA restriction analysis (ARDRA) and BOX-PCR analysis based genotyping clustered the isolates into different groups. Finally, four isolates were selected for plant growth promotion study in two tea commercial cultivars TV-1 and Teenali-17 in nursery conditions. The plant growth promotion study showed that the inoculation of consortia of these four PGPR isolates significantly increased the growth of tea plant in nursery conditions. Thus this study underlines the commercial potential of these selected PGPR isolates for sustainable tea cultivation.

## Introduction

Tea [*Camellia sinensis* (L.) O. Kuntze, family *Theaceae*] is an economically important, non-alcoholic caffeine-containing beverage crop cultivated for its leaf. India is currently the foremost producer, consumer and exporter of commercial tea. Tea is grown in over 36, 91,938 ha with an annual production of 4066.60 million kg globally, and India contributes 28% of world tea production with 21% of the area covered under tea cultivation [1]. The major tea growing areas in India are Assam, Darjeeling, the Nilgiri and other places in South India. The quality, flavour, aroma and other important traits of Darjeeling tea are primarily attributable to its geographical origin. Darjeeling district is located within the lesser and Sub-Himalayan belts of the Eastern Himalayas at an elevation of 6,700 ft. The Darjeeling hill area represents a unique geo-environmental perception and tea is grown here at up to 2000 m above mean sea level. In Darjeeling, tea is cultivated in over 17,820 ha with annual production of 8.91 million kg.

Tea plants are perennials and are mostly grown in a warm and humid climate, and their peculiar cultivation condition makes them more disease prone [2]. For the control of tea disease, chemicals are very commonly used in tea fields, which also cause environmental pollution and resistance among the target organisms. Therefore, microbial biological control has drawn attention and has been considered as a potential alternative to chemicals inputs [3]. In addition, the extensive use of chemicals such as fertilizers, fungicides and pesticides in tea fields for a prolonged period of time deteriorated the tea production and also reduced the soil fertility [4, 5]. Therefore, there is a need for eco-friendly alternative approach which can reduce the use of chemical application. However, the plant growth promoting rhizobacteria (PGPR) are considered as one of the most promising among the soil microorganisms to enhance plant health and growth rate without environmental contamination [6, 7].

The PGPR inoculants have been developed and applied in different agricultural crops like chilli [8], lentil [9], common bean [10], rice [11], tomato [12] and pepper [13] as biocontrol and biofertilizer agents. Although, the genetic diversity of PGPR has been reported in different crops such as wheat [14, 15], common bean [16], rice [17] and maize [18]. Hence, the study of PGPR is relatively advanced in different agricultural systems. Though, the PGPR associated with tea rhizosphere soil was reported previously [4, 5, 19, 20], however, research on PGPR associated with the tea crop still requires much further study. Hence, understanding and utilization of the root associated bacteria with the aim of growth promotion and sustainable tea cultivation in tea growing areas is important. In this study, the molecular techniques have been applied to investigate the genetic diversity of tea PGPR. PCR-based genotyping methods such as amplified ribosomal DNA restriction analysis (ARDRA) and amplification of repetitive extragenic palindromic-PCR (rep-PCR) like BOX-PCR are the strong suitable tools to evaluate microbial diversity in wide environmental microbial samples [21].

In the present study, an attempt was made to explore the functional and genetic diversity of culturable rhizobacteria in seven distinctly located tea estates of Darjeeling. The efficacy of the PGPR isolates was evaluated by plant growth promotion experiment on two different tea clones in nursery conditions. This study underlines the importance of the efficient plant growth enhancing rhizobacterial population associated with commercially cultivated tea plant in Darjeeling district, West Bengal, India. Microbial based bio-formulation containing this sort of PGPR can be used for sustainable tea cultivation in tea growing areas.

## Materials and methods

### Site description

Darjeeling is a small district located in the extreme north of the Indian state of West Bengal. It is situated in the Sub-Himalayan belt of the Eastern Himalayan range at an elevation of 6,700 ft. The soils in this region are mainly mixed sandy loam and silty. The temperature during summer reaches 18°C-20°C on the ridge and it drops up to 7°C-5°C during winter. The total annual rainfall in tea growing areas ranges from 1700 to 2500 mm with high humidity, dense fog and mist.

### Sample collection and isolation of tea rhizobacteria

**Fig 1:**
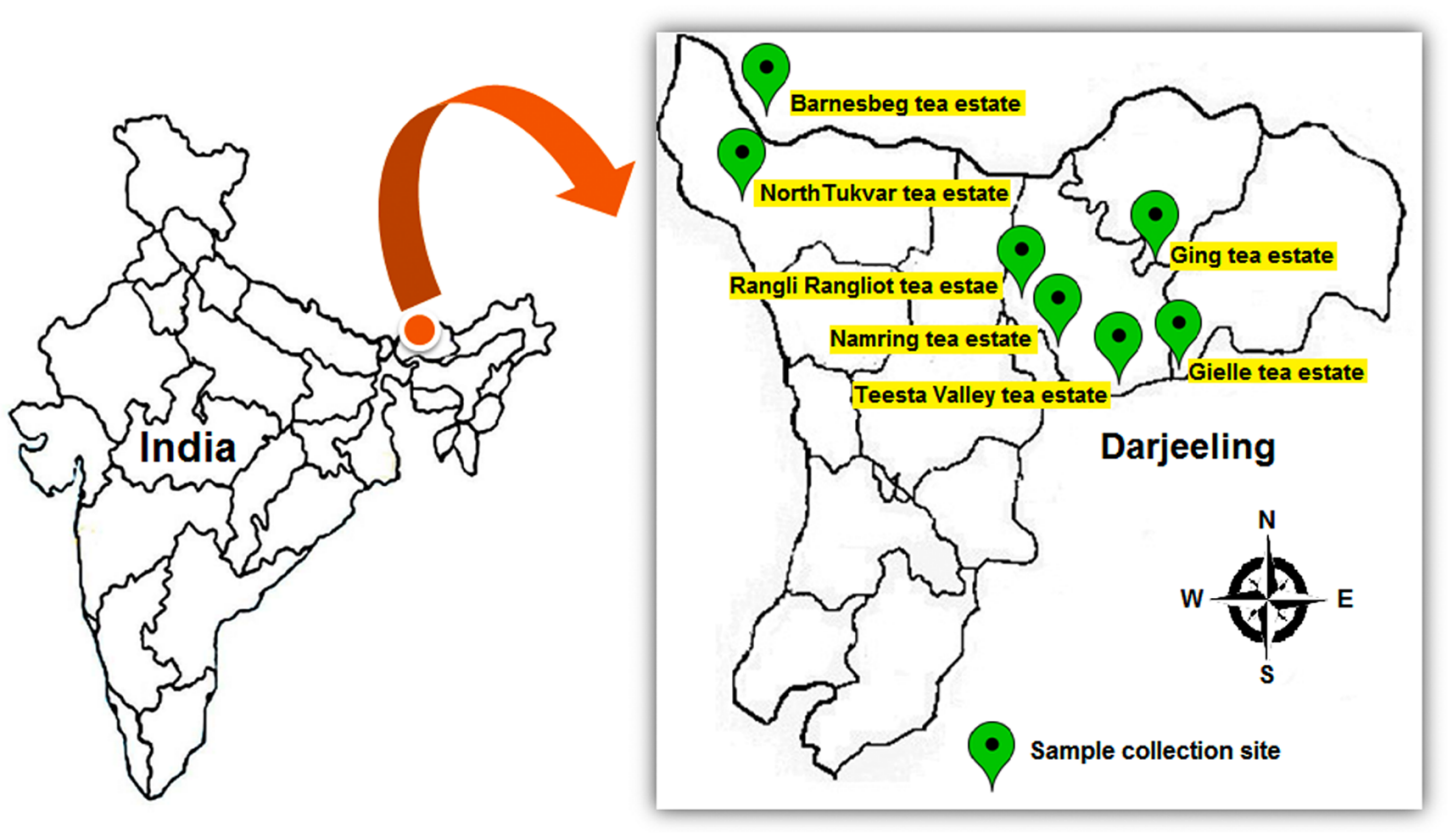
Map showing the location of sample collection sites.

All the rhizospheric soil samples of tea plants were collected in May, 2013 from seven different tea estates of Darjeeling, India i.e Gielle tea estate (27°00′52.27″N and 88°23′10.47″E, Elevation 871 m), Teesta Valley tea estate (27°00′44.55″N and 88°24′16.92″E, Elevation 1006 m), Barnesbeg tea estate (27°06′10.63″N and 88°15′53.30″E, Elevation 850 m), Rangli-Rangliot tea estate (27°01′27.60″N and 88°21′17.90″E, Elevation 1336 m), Namring tea estate (27°00′50.83″N and 88°22′12.24″E, Elevation 1011 m), North-Tukvar tea estate (27°05′42.53″N and 88°15′29.83″E, Elevation 975 m) and Ging tea estate (27°04′30.23″N and 88°17′58.67″E, Elevation 1066 m) as shown in Fig 1. For sample collection and isolation of rhizobacteria, we followed the same methodology as described in our previous work [20]. Soil samples were collected from rhizosphere zone about 5–25 cm depth. The area of the tea estates was divided into five blocks, from each block the samples were collected from five randomly selected healthy tea plants containing roots and root-adhered soil. Then, the five root soil samples from each block were pooled into one composite sample, resulting to a total of five composite samples. The soil samples were collected in sterile plastic bags and carried to the laboratory in an ice box.

The collected soil samples were processed for isolation of rhizobacteria immediately after reaching the laboratory. Rhizobacterial isolation was carried out by the conventional serial dilution method where 1 g of the soil with roots was suspended in 100 mL of saline solution (NaCl, 9 gL^-1^) and then kept at 30°C for 30 min with shaking (200 rpm) in an orbital shaker (New Brunswick Scientific, USA). The soil was then serially diluted up to 10^−6^ dilution using sterile saline. The samples were then agitated at maximum speed using vortex and an aliquot of 100 μL of each dilution was evenly spread plated over the surface of isolation media such as nutrient agar (NA), *Pseudomonas* agar (PA), *Azotobacter* media (AM) and *Azospirillum* agar (AS) procured from HiMedia, Mumbai, India. Plates were incubated at 30°C for 12–48 h and rhizobacterial colonies appeared on the isolation plates were counted. The population density of the rhizobacteria was measured by determining the number of colony forming unit (CFU) or total bacteria per gram of rhizosphere soil (Table 1). The morphologically distinct bacterial colonies were selected from isolation plates based on the bacterial colony morphology such as shape, size, elevation, opacity and margin. The selected bacterial isolates were subcultured to purify and maintained in 15% glycerol vials, in -80°C for future use. Gram staining of the bacterial isolates was performed and observed under light microscope (40X, Motic BA410 trinocular Microscope).

**Table 1:** Location, soil type and number of bacteria isolated from tea rhizosphere soil samples collected from the tea estate of Darjeeling.

Tea estates	Location	Soil type (USDA)	pH	CFU g^-1^ range	Number of isolates
Gielle tea estate	27°00′52.27″N 88°23′10.47″E, Elevation 871 m	Sandy loam	4.3	6×10^4^−3 ×10^6^	20
Teesta Valley tea estate	27°00′44.55″N 88°24′16.92″E, Elevation 1006 m	Sandy loam	4.1	5×10^4^−2×10^6^	21
Barnesbeg tea estate	27°06′10.63″N 88°15′53.30″E, Elevation 850 m	Sandy loam	4.5	4×10^4^–3.2×10^6^	20
Rangli-Rangliot tea estate	27°01′27.60″N 88°21′17.90″E, Elevation 1336 m	Sandy loam and clay	4.2	4×10^4^−1.2 ×10^6^	15
Namring tea estate	27°00′50.83″N 88°22′12.24″E, Elevation 1011 m	Sandy loam	4.4	4×10^4^−2 ×10^6^	18
North-Tukvar tea estate	27°05′42.53″N 88°15′29.83″E, Elevation 975 m	Sandy loam, silty	4.6	8×10^4^−5 ×10^6^	32
Ging tea estate	27°04′30.23″N 88°17′58.67″E, Elevation 1066 m	Sandy loam	4.2	4×10^4^−2 ×10^6^	24

### Fungal cultures

The test fungal pathogens *Pestalotiopsis theae* (ITCC 6599), *Curvularia eragrostidis* (ITCC 6429), *Glomerella cingulata* (MTCC 2033), *Rhizoctonia solani* (MTCC 4633) and *Fusarium oxysporum* (MTCC 284) were used in this study. Foliar fungal pathogen *Nigrospora sphaerica* (KJ767520) reported from Darjeeling tea plantation [22] was also used as test pathogen. The fungal pathogens were cultured in potato dextrose agar (PDA) medium at 25°C. The fungal pathogens were procured from the Microbial Type Culture Collection (MTCC), Institute of Microbial Technology, Chandigarh, India and Indian Type Culture Collection (ITCC), Indian Agricultural Research Institute, New Delhi, India.

### *In vitro* screening of tea rhizobacteria for antifungal activity

The rhizobacterial isolates were subjected to evaluation of their antagonistic activity against the tea pathogenic fungi *P*. *theae*, *N*. *sphaerica*, *C*. *eragostidis*, *G*. *cingulata*, *R*. *solani* and *F*. *oxysporum* on PDA medium. The bacterial broth cultures were adjusted to 1×10^8^ CFU mL^-1^ and a loopful of bacterial cultures was streaked equidistantly on the edge of the PDA plates. The fungal agar plug (5 mm) of the test fungal mycelium grown on the PDA plate was placed at the centre of the plate between the bacterial streaked lines. The plates were incubated at 28±2°C for 5 days. Control plates were prepared with the fungal agar plug without the bacterial streaks. The antagonistic activity was evaluated by comparing the fungal mycelial diameter on control and test plates, and the percentage of inhibition was calculated by using the formula C-T/C×100, where C and T are the fungal mycelial diameter on control and the test plate respectively [23].

### *In vitro* screening for antifungal traits

#### Siderophore production

The detection of siderophore production was done by using Chrome Azurol S (CAS) agar medium as described previously [24]. The siderophore was developed by forming the ternary complex chrome azurol S/Fe(III)/hexadecyltrimethylammonium bromide as an indicator and formed orange halos around the bacterial colonies. The quantification of siderophore was carried out by the method described by Patel et al. [25] in which 0.5 mL of culture supernatant was mixed with 0.5 mL of CAS reagent. The experiment was performed with three replicates. The uninoculated medium was considered as reference and the absorbance was read in the multimode reader (Varioskan flash, Thermo Scientific, USA) at 630 nm. Siderophore content in the sample was calculated by using the following formula:

%siderophore units=Ar−As/Ar×100

Where, Ar and As are absorbance of reference (CAS reagent) and absorbance of sample at 630 nm.

#### Activity of chitinase, cellulase and protease of rhizobacterial isolates

The bacterial isolates were screened for the chitinase production by spot plate assay. For chitinase assay, colloidal chitin was prepared as described previously [26]. The nutrient broth (NB) media amended with 1% colloidal chitin was prepared for chitinase assay. The bacterial isolates were spotted on the plates and incubated at 30°C for 48 h to observe the clear zones around the bacterial colonies. The clear zones showed around the bacterial colonies were considered as positive chitinase activity. The cellulase activity was screened by spot inoculation of bacterial isolates on carboxymethylcellulose (CMC) agar medium. The plates were incubated at 30°C for 48 h. The gram’s iodine solution was poured on the CMC plates to observe the clear zones around the bacterial colonies [27]. The isolates showed the clear zones were considered as positive for cellulase activity. The extracellular protease production was screened by skim milk agar. The bacterial isolates were spotted on the surface of the medium. The protease production was determined by the formation of the clear zone surrounding the bacterial isolates due to the breakdown of milk protein [28]. The isolates showed the clear zones were considered as positive for protease activity.

### *In vitro* screening for plant growth promoting (PGP) traits

The rhizobacterial isolates were screened for a wide array of PGP traits. Indole acetic acid (IAA) production was tested using Salkowski’s reagent as described by Gordon and Weber [29]. Phosphate solubilisation was determined using the protocol as previously described [30]. The bacterial isolates that formed halos around their colonies were considered as phosphate solubilizing strains. Ammonia production was tested as previously described [31]. The rhizobacterial isolates were also screened for ACC deaminase (1-Aminocyclopropane-1-carboxylate deaminase) activity by using Dworkin and Foster (DF) minimal salts media [32] supplemented with 3 mM ACC as the sole nitrogen source [33].

### Quantitative assay for PGP traits

#### IAA production

The quantification of IAA was done by inoculation of the purified rhizobacterial isolates in M9 glucose minimal salts media [34] supplemented with L-tryptophan (100 μg mL^-1^). The L-tryptophan solution was filter-sterilized by using 0.2 μm membrane filters. The bacterial cultures were grown for 72 h and harvested by centrifugation (12000 rpm for 15 min at 4°C). To estimate IAA production, the bacterial supernatant was mixed with the Salkowski’s reagent (48 mL 35% HClO_4_ containing 2 mL 0.5 M FeCl_3_) in 1:2 ratio (bacterial supernatant: reagent) at room temperature. After 25 min, the mixture was quantified at 530 nm in 96 well microtiter plates by using the multimode reader (Varioskan flash, Thermo Scientific, USA) with three replicates [29]. Development of pink or red colour indicates IAA production. The concentration of IAA produced by the bacterial isolates was compared by extrapolating in the standard curve prepared by using commercial IAA at different concentrations (Sigma, Aldrich, USA).

#### Phosphate solubilization

The estimation of tri-calcium phosphate solubilisation in Pikovskaya's liquid medium was carried out with slight modification as described by Fiske and Subbarow [35] using ammonium molybdate reagent. The absorbance was measured at 650 nm in three replicates using the multimode reader (Varioskan flash, Thermo Scientific, USA) and the degree of phosphate solubilisation of each isolate was obtained by using the calibration curve prepared by using KH_2_PO_4_. The variation in pH of Pikovskaya's medium during the growth of each isolate was observed.

#### Ammonia production

The bacterial isolates were grown in the peptone water broth for 48–72 h at 30°C. After centrifugation, the bacterial supernatant was mixed with Nessler’s reagent. Development of brown to yellow colour indicates the presence of ammonia. The absorbance was measured at 450 nm using the multimode reader (Varioskan flash, Thermo Scientific, USA) with three replicates. To estimate the amount of ammonia production by the isolates, a standard curve of ammonium sulphate was prepared [36].

### Plant growth promotion assay in nursery conditions

The plant growth promotion experiment in nursery conditions was carried out in Kopati tea estate nursery which is located in Darrang district, Assam, India during 15^th^ July 2015 to 20^th^ January 2016. The experiment was conducted in two different types of tea clones used for commercial cultivation i.e. TV-1 (Assam-China hybrid standard clone) and Teenali-17 released by Tocklai Tea Research Institute (TTRI), Jorhat, Assam, India. Six months old tea clones of approximately equal sizes were selected for this experiment and the clones were maintained in polyethylene sleeves of size 15–17.7 cm layflat, 20–25 cm long and 150 gages thick. The experiment was set up in completely randomized block design. Each block had total of 30 plants in three replicates i.e., one replicate had 10 tea plants. Thirteen blocks were made for each tea clone i.e. total 26 blocks were made for two tea clones. There were 13 different treatments for 13 blocks including one block for commercial fertilizer and one block for the untreated control plants.

The selected bacterial inoculums were grown on nutrient broth (HiMedia, India) for 48 h and 180 rpm continuous shaking at 30°C. The bacterial cells were centrifuged at 12,000 rpm for 15 min and the pellets were diluted with sterile distilled water to make a final concentration of 10^8^−10^9^ CFU mL^-1^. The resulting bacterial cell suspensions, 100 mL/plant were used as a soil drench [4, 5] to treat tea plants under nursery conditions only once. The soil of the tea sleeves was non-sterile. The plants were harvested after 6 months of inoculation. Shoot and root length, fresh and dry shoot and root weight and the number of tea leaves were compared with the commercial fertilizer and uninoculated control plants. Fresh shoots and roots weight was immediately taken after the harvest of the tea plants. The roots and shoots were completely dried under hot air oven at 50°C for few days to take the dry roots and shoots weight. The weight of the dry roots and shoots were measured every day until there was no significant weight difference observed. The treatment details for PGP assay in nursery condition are as follows:

### Treatment details

Control: Sterile nutrient broth medium without bacterial inoculums
CF: Commercial fertilizer (N:P:K in 2:1:2 mixture)*
T1: Treatment 1 (strain TTD5)
T2: Treatment 2 (strain TTD21)
T3: Treatment 3 (strain BT22)
T4: Treatment 4 (strain NT5)
T5: Treatment 5 (strain TTD5+ strain TTD21)
T6: Treatment 6 (strain TTD5+ strain BT22)
T7: Treatment 7 (strain TTD5+ strain NT5)
T8: Treatment 8 (strain TTD21+ strain BT22)
T9: Treatment 9 (strain TTD21+ strain NT5)
T10: Treatment 10 (strain BT22+ strain NT5)
T11: Treatment 11 (strain TTD5+ strain TTD21+ strain BT22 + strain NT5)

*Young tea dose of 20 g/tree was applied as recommended by TTRI, Jorhat, Assam India “Field Management Notes”.

### Molecular characterization and phylogenetic analysis

#### DNA extraction and 16S rRNA gene amplification

Genomic DNA of rhizobacterial isolates were extracted by using QIAamp DNA mini kit (Qiagen, India) according to manufacturer’s instructions and analyzed by 0.8% agarose gel. The purity and quantity of the DNA were checked in Nanodrop spectrophotometer (Thermo Scientific, USA). The DNA yield was quantified in ng mL^-1^ and the purity was assessed at a 260/280 nm absorbance ratio. The PCR amplification of 16S rRNA gene was performed using universal primers PA (5′-AGAGTTTGATCCTGGCTCAG-3′) and 1492R (5′-GGTTACCTTGTTACGACTT-3′) as previously described [37].

#### Amplified Ribosomal DNA Restriction Analysis (ARDRA)

The 16S rRNA gene PCR product was purified by GenElute PCR clean up kit (Sigma-Aldrich, USA) and 20 μL of the purified PCR products were digested by 1.5 U of three different restriction enzymes *Hae*III, *Msp*I, *Hinf*I (New England Biolabs, UK) according to manufacturer’s instruction and incubated for 3 h at 37°C. The resulting digested fragments together with 100 bp marker (Merck Genei, India) were resolved by gel electrophoresis at 60 V on 2% agarose gels in 1XTAE buffer containing 10 μg mL^-1^ ethidium bromide. After electrophoresis, the gel was visualized under gel documentation system (Bio-Rad, USA) and analysed by software NTSYS-pc version 2.02. (Applied Biostatistics Inc., New York).

#### BOX-PCR fingerprinting

The genotypic analysis of bacterial isolates by rep PCR using BOX-A1R primer (5′-CTACGGCAAGGCGACGCTGACG-3′) was carried out as described previously [38]. The PCR product along with 500 bp DNA ladder (Merck Genei, India) was subjected to electrophoresis using 2% agarose gel stained with 10 μg mL^-1^ ethidium bromide in 1XTAE buffer. The gels were observed under the gel documentation system (Bio-Rad, USA) and the generated fingerprints were analysed by hierarchical clustering using Phoretix 1D Pro gel analysis software (TotalLab Ltd, Newcastle upon Tyne, England).

#### Phylogenetic analysis

The rhizobacterial isolates from each representative clade were selected based on the BOX-PCR and ARDRA analysis. The 16S rRNA gene for the selected isolates was sequenced by Scigenom sequence service provider (Scigenom Labs Pvt. Ltd., Cochin, India). The nucleotide sequences were compared against nucleotide databases using the NCBI BLASTn and EzTaxon server 2.1 programs to identify the closest known taxa [39]. The 16S rRNA gene along with their closest homology sequences were aligned using multiple sequence alignment program CLUSTAL W algorithm implemented in MEGA 6 software by using default parameters [40]. The phylogenetic tree was constructed by neighbour-joining (NJ) method using MEGA 6 program and evolutionary distances were computed with the help of Kimura's 2 parameter models [41]. The bootstrap analysis with 1000 replications using *p*-distance model was performed based on the original dataset to estimate the confidence of a particular clade [42]. The 16S rRNA gene sequences obtained in the present study was submitted to GenBank under accession numbers KX373959-KX373991.

### Detection of chitinase gene of potential chitinase producing strains

The glycoside hydrolase family 18 group A bacterial chitinase gene was amplified by using degenerate primer pair GA1F and GA1R as previously described [43]. The 10 μL of PCR reaction mixture contained 1 μL of 10X Taq DNA buffer, 2.5 mM dNTP mix, 0.2 μM of primers, 1U *Taq* DNA polymerase and 10 ng of bacterial genomic DNA. The amplification was carried out in proflex PCR system (Applied Biosystems, USA) using the following conditions: initial denaturation at 95°C for 5 min; 35 cycles of 1 min at 95°C, 30 sec at 60°C and 1 min at 72°C; final extension at 72°C for 7 min. The amplified product was resolved on 2% agarose gel in 1X TAE buffer and visualized under the gel documentation system (Bio-Rad, USA).

### Data analysis

The isolates showing antagonistic activity against the different fungal pathogens were represented as Venn diagram using the multiple dataset analysis features of Vennture software [44]. The Shannon-Wiener diversity index (H) along with the evenness index (E) was calculated [45]. The means and standard deviations of the data were calculated. Analysis of variance (ANOVA) was performed in SPSS package (SPSS 18.0, SPSS Inc., Chicago, IL, USA) to study the level of significance for tea plant growth promotion experiment in nursery conditions. The significance of effects of the treatments was determined based on Duncan's multiple range tests at *P* < 0.05. Principal component analysis (PCA) was performed on the datasets to evaluate the relationship between the samples to analyze the effect of bacterial treatment on plant growth promotion. The PCA was performed by MetaboAnalyst 3.0 software [46].

## Results

### Isolation of rhizobacteria from tea rhizosphere soil

A total of 150 morphologically distinct rhizobacteria were isolated from seven different tea estates of Darjeeling using different isolation media. The population density of rhizobacterial isolates in tea rhizosphere soil ranged from 6×10^4^ to 3×10^6^ and the total number of isolates from each tea estate was enumerated (Table 1). The pH of the soil samples was measured and the texture of the soil samples identified according to USDA soil classification [47].

### *In vitro* antagonistic activity against fungal pathogens

**Fig 2:**
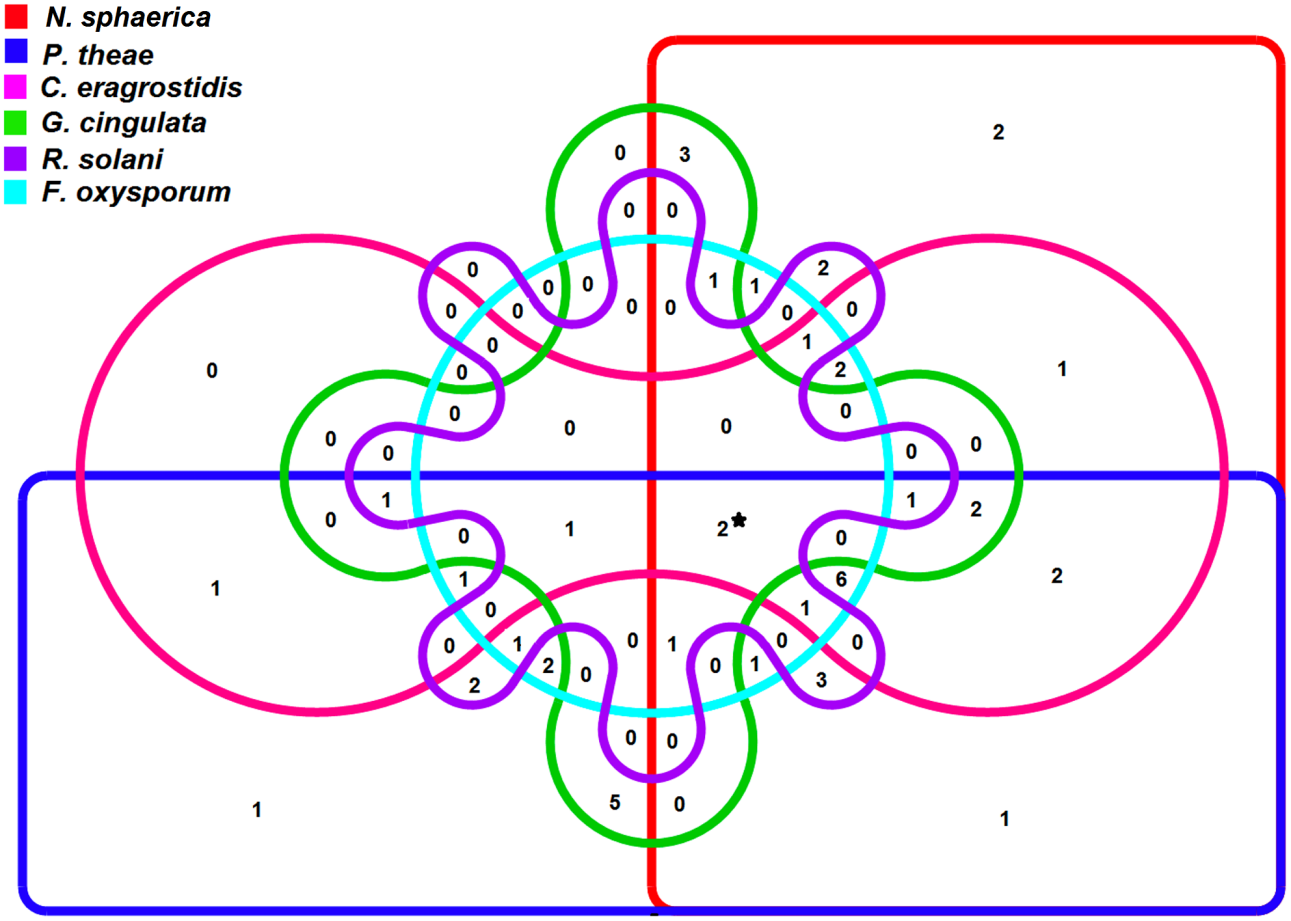
Venn diagram representation of rhizobacterial isolates showing antagonistic activity against fungal pathogens. The Venn diagram shows the distribution of 48 antagonistic rhizobacterial isolates into 6 profiles which are representing the 6 test fungal pathogens. (*2 isolates showed antagonistic activity against all the test fungal pathogens) (See S1 Table).

All 150 rhizobacterial isolates obtained were screened for the production of antifungal activity against six fungal pathogens. Forty eight (32%) isolates exhibited antagonistic activity against at least one of the test pathogens (Fig 2 and S1 Table). Out of the 48 isolates, 33 (68.7%) isolates showed antagonism against *N*. *sphaerica*, 35 (72.9%) isolates showed activity against *P*. *theae*, 22 (45.8%) isolates showed activity against *C*. *eragrostidis*, 17 (35.4%) isolates showed activity against *G*. *cingulata*, 21 (43.7%) isolates showed activity against *R*. *solani* and 18 (37.5%) isolates showed activity against *F*. *oxysporum*. In addition, 15 (31.3%) isolates showed antagonistic activity against any four pathogens. Only two isolates (strains GN14 and TTD7) showed antagonistic activity against all the pathogens. These 48 isolates were further evaluated by subsequent *in vitro* experiments.

### Antifungal and plant growth promoting (PGP) traits of antagonistic rhizobacteria

#### Quantitative estimation of siderophore production

The 48 rhizobacterial isolates showed siderophore production in the range from 12% to 45.6% of siderophore units. Only 11 (22.9%) isolates were able to produce 30% or more siderophore units and 5 isolates produced more than 40% units of siderophore. Isolate TV1 showed the highest production of siderophore units i.e. 45.6% (Table 2).

**Table 2:** Screening of selected rhizobacteria for PGP and antifungal traits.

Sl No	Strain code	PGP traits				Antifungal traits				
		IAA production (μg mL^-1^)*	Phosphate solubilization (μg mL^-1^)*	Ammonia production (μmol mL^-1^)*	ACC deaminase activity	Siderophore production (%)*	Chitinase activity	Cellulase activity	Protease activity	Chitinase gene
1	TTD1	21.2 ± 0.2	107.1 ± 0.1	2.3 ± 0.1	**+**	22.9± 0.3	**+**	**-**	**-**	**+**
2	TTD2	15.2 ± 0.2	176.8 ± 0.1	5.6 ± 0.3	**+**	28.5% ± 0.5	**-**	**-**	**-**	**-**
3	TTD3	13.7 ± 1.4	108.9 ± 0.1	5.6 ± 0.1	**+**	20% ± 2.4	**-**	**-**	**+**	**-**
4	TTD5	20.6 ± 0	187.3 ± 0.2	5.4 ± 0.1	**+**	45.3± 0.3	**+**	**-**	**-**	**+**
5	TTD7	73.1 ± 0.2	187.7 ± 0.1	6 ± 0.2	**+**	37.7% ± 0	**+**	**-**	**+**	**+**
6	TTD8	28.1 ± 0.8	67.2 ± 0.2	5.7 ± 0.2	**-**	12% ± 0.3	**-**	**+**	**-**	**-**
7	TTD10	16.4 ± 0.2	121.4 ± 0	5.9 ± 0.1	**+**	22.8% ± 0.2	**-**	**+**	**-**	**-**
8	TTD14	33.3 ± 0.2	155.9 ± 0.1	5.1 ± 0.2	**+**	24.7% ± 0.9	**-**	**+**	**-**	**-**
9	TTD15	37.6 ± 1.9	112.5 ± 0.1	5.9 ± 0.1	**+**	44.5% ± 2.4	**-**	**+**	**-**	**-**
10	TTD16	17.2 ± 0.7	68 ± 0	5.9 ± 0.2	**+**	22.9% ± 0.3	**-**	**-**	**+**	**-**
11	TTD19	57.8 ± 2	242.7 ± 0.2	4.5 ± 0.2	**+**	22.2% ± 0.3	**+**	**-**	**-**	**+**
12	TTD21	11.4 ± 0.8	89.3 ± 0.1	6 ± 0	**+**	23.3% ± 0.3	**-**	**+**	**-**	**-**
13	BT2	24.6 ± 1.6	175.2 ± 0.1	5.7 ± 0.1	**-**	24.5% ± 2.5	**-**	**-**	**+**	**-**
14	BT4	18.6 ± 0.4	47.9 ± 0.1	5.8 ± 0.2	**-**	23.5% ± 1.3	**-**	**-**	**-**	**-**
15	BT6	28.7 ± 1.4	158.3 ± 0.1	6 ± 0.3	**-**	30.2% ± 1	**+**	**-**	**+**	**+**
16	BT13	13.4 ± 0.2	123.6 ± 0.1	5.9 ± 0.4	**-**	18.8% ± 1.1	**-**	**-**	**+**	**-**
17	BT15	27.6 ± 2.2	143.8 ± 0.1	5.3 ± 0.1	**+**	28.8% ± 1.1	**+**	**-**	**+**	**+**
18	BT19	23.9 ± 0.8	119.2 ± 0.1	5.9 ± 0.4	**+**	35.5% ± 3.3	**-**	**-**	**-**	**-**
19	BT20	14.6 ± 1.6	57.2 ± 0.1	5.8 ± 0.3	**+**	25.7% ± 1.4	**-**	**-**	**+**	**-**
20	BT22	29.8 ± 0.3	102.2 ± 0	5.8 ± 0.2	**+**	21% ± 2.4	**-**	**+**	**+**	**-**
21	GN2	17.3 ± 0.3	113.7 ± 0.1	5.6 ± 0.2	**+**	26% ± 2.9	**-**	**+**	**+**	**-**
22	GN6	26.6 ± 0.1	76.2 ± 0.2	5.4 ± 0.2	**+**	43.3% ± 2.3	**-**	**-**	**+**	**-**
23	GN9	12.8 ± 0.8	181.4 ± 0.1	5.5 ± 0.2	**+**	18.1% ± 0.1	**-**	**+**	**-**	**-**
24	GN10	26.3 ± 0.2	89.2 ± 0	5.3 ± 0.1	**+**	18.5% ± 0.5	**-**	**+**	**-**	**-**
25	GN14	47.7 ± 2.1	254.7 ± 0.1	5.5 ± 0.4	**+**	39.7% ± 2.3	**+**	**-**	**+**	**+**
26	GN17	15.1 ± 0	122.9 ± 0.1	5.5 ± 0.4	**+**	18.5% ± 0.5	**-**	**+**	**+**	**-**
27	GT4	12.8 ± 1	115.9 ± 0	6 ± 0	**+**	17.9% ± 1.3	**+**	**-**	**-**	**+**
28	GT7	37.2 ± 0.6	245.9 ± 0.1	6 ± 0.1	**+**	27.4% ± 0.8	**-**	**+**	**+**	**-**
29	GT12	16.4 ± 0.3	230.7 ± 0.2	5.4 ± 0.2	**+**	18.1% ± 0.1	**-**	**+**	**-**	**-**
30	GT15	14.2 ± 0.8	137.2 ± 0.1	5.7 ± 0.5	**+**	18.1% ± 0.1	**+**	**-**	**+**	**+**
31	GT20	36.1 ± 0.8	180 ± 0.1	5.8 ± 0.2	**+**	27.9% ± 0.1	**+**	**+**	**-**	**+**
32	GT22	9.9 ± 0.8	166.5 ± 0.1	5.8 ± 0.3	**+**	22.2% ± 0.3	**+**	**-**	**+**	**+**
33	GT32	34.3 ± 1.7	204.9 ± 0.1	5.4 ± 0.3	**+**	20% ± 0.9	**+**	**+**	**+**	**+**
34	GT33	31.8 ± 0.1	120.8 ± 0	6 ± 0.1	**+**	18.3% ± 2.4	**-**	**-**	**-**	**-**
35	GT34	26.3 ± 0.3	167.7 ± 0.1	5.4 ± 0.4	**+**	22.8% ± 0.2	**-**	**-**	**-**	**-**
36	GT35	15.1 ± 0	100.8 ± 0.1	5.9 ± 0.2	**+**	22.5% ± 0.2	**-**	**-**	**-**	**-**
37	TV1	57.9 ± 0.5	186.8 ± 0	5.5 ± 0.2	**+**	45.6% ± 1.6	**+**	**+**	**+**	**+**
38	TV2	14.6 ± 0.7	97.1 ± 0.1	5.4 ± 0.4	**-**	21% ± 0.4	**-**	**+**	**+**	**-**
39	TV7	26.9 ± 0.8	125.4 ± 0.1	5.5 ± 0.4	**-**	31.9% ± 0.1	**-**	**-**	**+**	**-**
40	TV9	26 ± 0.8	173 ± 0.1	5.5 ± 0.4	**-**	28.7% ± 0.3	**-**	**-**	**-**	**-**
41	TV13	13.1 ± 0.1	124.2 ± 0	5.5 ± 0.2	**-**	23.6% ± 0.9	**-**	**+**	**+**	**-**
42	TV15	18.1 ± 0.2	74.9 ± 0	4.5 ± 0.2	**-**	23.4% ± 0.3	**-**	**-**	**-**	**-**
43	NT4	34.4 ± 0.1	234.7± 0.1	6.2 ± 0.2	**+**	33.8% ± 0.7	**+**	**-**	**+**	**+**
44	NT5	26.8 ± 0.7	124 ± 0.1	4.3 ± 0.3	**-**	21.7% ± 0.6	**-**	**-**	**-**	**-**
45	NT8	12.8 ± 0.8	65.5 ± 0	5.5 ± 0.4	**+**	24% ± 0.7	**-**	**-**	**-**	**-**
46	RR10	12.6 ± 1.2	132.3 ± 0	3.8 ± 0.1	**+**	18.1% ± 0.4	**-**	**-**	**+**	**-**
47	RR14	29 ± 0	177.8 ± 0	4.8 ± 0.2	**-**	18.1% ± 0.3	**-**	**-**	**+**	**-**
48	RR16	34.1 ± 0.2	117.7 ± 0.1	5.7 ± 0.2	**+**	17.8% ± 0.7	**-**	**+**	**+**	**-**

*Mean value (all values are triplicate) ± Standard deviation (SD), + means positive activity and—means no activity

#### Quantitative estimation of IAA production, phosphate solubilization and ammonia production

Out of the 48 isolates, 12 (25%) isolates showed 30 μg mL^-1^ or more IAA production and isolate TTD7 showed the maximum IAA production (73.1 μg mL^-1^). Most of the isolates exhibited good phosphate solubilization and 6 isolates showed phosphate solubilization up to 200 μg mL^-1^ or more. Amongst all, isolate GN14 showed the highest phosphate solubilization with 254.7 μg mL^-1^. The ammonia production was observed in the range of 2.3 μmol mL^-1^ to 6.2 μmol mL^-1^ and most of the isolates showed 5 μmol mL^-1^ or more ammonia production. Isolate NT4 produced maximum ammonia with 6.2 μmol mL^-1^ among all the isolates (Table 2).

#### Screening of rhizobacteria for chitinase, protease, cellulase and ACC deaminase production

All the 48 isolates were screened for *in vitro* chitinase, protease, cellulase and ACC deaminase production. Fourteen (29%) isolates showed chitinase production and these isolates also showed the presence of glycoside hydrolase family 18 group A bacterial chitinase gene of size around 400 bp in PCR amplification (S1 Fig). Moreover, 18 (38%) isolates showed cellulase production, 25 (52%) isolates showed protease production and 36 (75%) isolates showed ACC deaminase activity (Table 2).

### Assessment of the potent rhizobacteria showing *in vitro* antifungal and PGP traits

To select the most promising isolates showing antagonistic activity along with PGP traits among the selected rhizobacteria, an assessment was made by using bonitur scale [23, 48]. In this scale, points are assigned for each *in vitro* bacterial trait tested and the maximum possible bonitur score is 34 points. For the antifungal activity, the maximum point is three for each fungal pathogen and in this study, six fungal pathogens were used for the antifungal activity, thus totalling to 18 points. Production of an antifungal trait like siderophore is assigned three points, and along with that chitinase production, protease production and cellulase production are evaluated with one point each totalling to 6 points. The PGP traits like IAA production, phosphate solubilization and ammonia production are maximum three points each and ACC production is one point totalling to 10 points. The summation of total assessment point greater than or equal to 15, out of 34 is taking as a cut off for qualifying as promising rhizobacterial isolates. The assessment showed that out of the 48 isolates screened, 15 isolates qualified as promising isolates (Table 3).

**Table 3:** The most promising 15 isolates and their antagonistic activity, antifungal mechanisms along with their PGP traits and general assessment and ranking for their ability to function as PGPR.

StrainCode	Antagonistic activities						Antifungal traits				PGP traits				Total Ass. (34)^o^	Rank
	GI percentage (%)															
	Ns^a^	Pt^b^	Ce^c^	Gc^d^	Rs^e^	Fo^f^	Sid^g^	Chi^h^	Pro^i^	Cel^j^	IAA^k^	PS^l^	Am^m^	ACC^n^		
GN14	3	2	3	3	3	3	3	1	1	0	2	3	3	1	31	1^st^
TTD7	3	2	3	3	2	3	3	1	1	0	3	2	3	1	30	2^nd^
TV1	3	3	2	0	1	2	3	1	1	1	2	2	3	1	25	3^rd^
TTD5	3	1	0	2	3	3	3	1	0	0	1	2	3	1	23	4^th^
GT7	0	2	3	2	0	2	2	0	1	1	2	3	3	1	22	5^th^
BT2	3	0	3	0	3	3	2	0	1	0	1	2	3	0	21	6^th^
NT4	2	1	3	0	2	0	3	1	1	0	1	3	3	1	21	6^th^
BT15	2	1	3	0	3	0	2	1	1	0	1	2	3	1	20	7^th^
GT20	2	1	2	0	3	0	2	1	0	1	2	2	3	1	20	7^th^
GT32	1	2	3	2	0	0	1	1	1	1	1	3	3	1	20	7^th^
TTD15	2	1	2	2	0	2	3	0	0	0	2	1	3	1	19	8^th^
GT34	3	1	3	3	0	0	2	0	0	0	1	2	3	1	19	8^th^
BT6	1	2	2	0	3	0	2	1	1	0	1	2	3	0	18	9^th^
TTD19	1	1	2	0	1	0	1	1	0	0	2	3	2	1	15	10^th^
TV9	0	1	2	1	1	2	2	0	0	0	1	2	3	0	15	10^th^

^a^Ns—*Nigrospora sphaerica*,

^b^Pt—*Pestalotiopsis theae*,

^c^Ce—*Curvularia eragrostidis*,

^d^Gc—*Glomerellacingulata*,

^e^Rs—*Rhizoctonia solani*,

^f^Fo—*Fusarium oxysporum*,

^g^Sid—Siderophore production,

^h^Chi—Chitinase production,

^i^Pro—Protease production,

^j^Cel—Cellulase production,

^k^IAA—Indole acetic acid production,

^l^PS—Phosphate solubilization,

^m^Am—Ammonia production,

^n^ACC—ACC deaminase production,

^o^Total assessment score, GI—Growth inhibition.

### Plant growth promotion assay in nursery conditions

**Fig 3:**
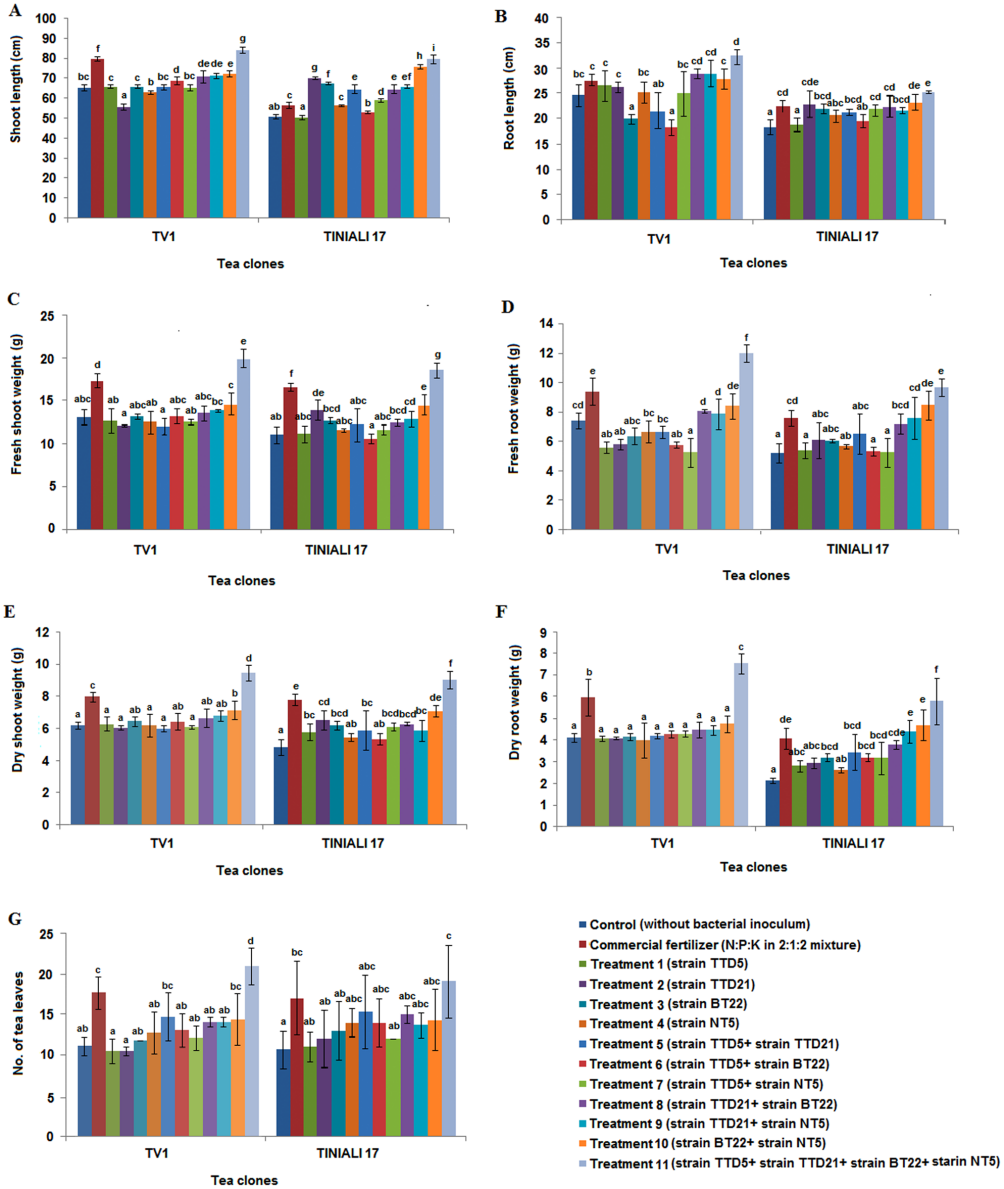
Evaluation of different PGP parameters to show the effect of treatments in two different varieties of tea clones TV-1 and Teenali-17in nursery conditions. (A) shoot length, (B) root length, (C) fresh shoot weight, (D) fresh root weight, (E) dry shoot weight, (F) dry root weight and (G) number of leaves. Values having different superscripts (a-i) differ significantly (*P* < 0.05). [Control (without bacterial inoculums), CF (N:P:K in 2:1:2 mixture), Treatment1 (strain TT5), Treatment 2 (strain TTD21), Treatment 3 (strain BT22), Treatment 4 (strain NT5), Treatment 5 (strain TTD5+ strain TTD21), Treatment 6 (strain TTD5+ strain BT22), Treatment 7 (strain TTD5+ strain NT5), Treatment 8 (strain TTD21+ strain BT22), Treatment 9 (strain TTD21+ strain NT5), Treatment 10 (strain BT22+ strain NT5) and Treatment 11 (strain TTD5+ strain TTD21+ strain BT22 + strain NT5)].

**Fig 4:**
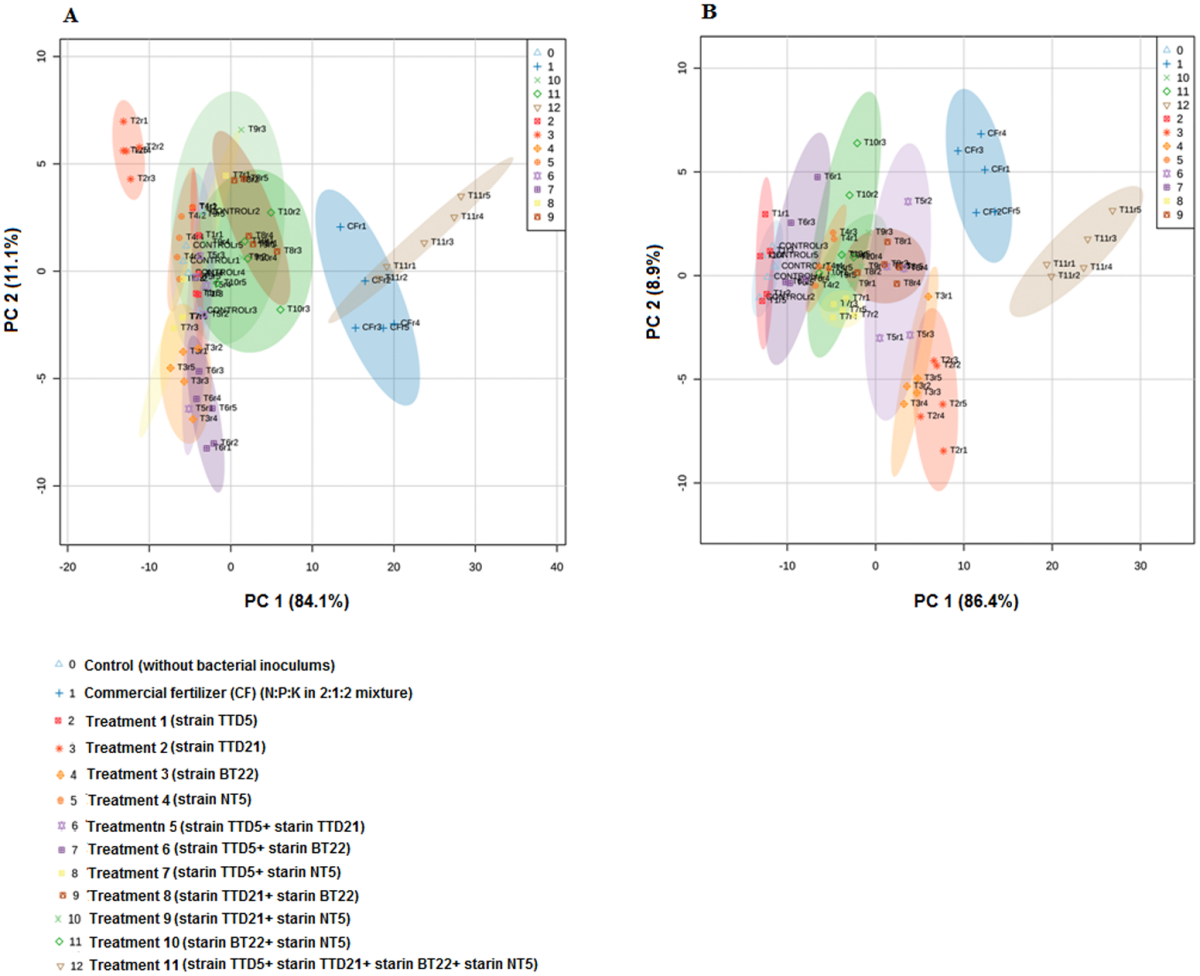
PCA analysis showed the effect of PGPR treatments on plant growth parameters of two different varieties of tea clones TV-1 and Teenali-17 in nursery conditions. The PCA analysis was performed taking the plant growth parameters of root and shoot length, fresh and dry roots and shoots weight and number of tea leaves. (A) and (B) showing the treatment 11 and commercial fertilizer (CF) clustered closer to each other which showed significantly increased in plant growth parameters than the control and other treatments. (The r1, r2, r3, r4 and r5 in the PCA plot are representing the five replications for each treatment). [Control (without bacterial inoculums), CF (N:P:K in 2:1:2 mixture), Treatment1 (strain TT5), Treatment 2 (strain TTD21), Treatment 3 (strain BT22), Treatment 4 (strain NT5), Treatment 5 (strain TTD5+ strain TTD21), Treatment 6 (strain TTD5+ strain BT22), Treatment 7 (strain TTD5+ strain NT5), Treatment 8 (strain TTD21+ strain BT22), Treatment 9 (strain TTD21+ strain NT5), Treatment 10 (strain BT22+ strain NT5) and Treatment 11 (strain TTD5+ strain TTD21+ strain BT22 + strain NT5)].

For plant growth promotion experiment in nursery, four isolates TTD5, TTD21, BT22 and NT5 were selected. There were several other bacterial strains that showed more promising *in vitro* activity than the selected isolates as showed in the assessment table. However, the 16S rRNA gene sequencing and similarity search by BLASTn and EzTaxon revealed that those bacterial species are generally considered as human opportunistic pathogens. Furthermore, the above mentioned isolates were identified as *Brevibacillus agri* strain TTD5, *Aneurinibacillus aneurinilyticus* strain TTD21, *Sporosarcina koreensis* strain BT22 and *Bacillus megaterium* strain NT5 where no such human infections were reported and were considered as non pathogenic isolates. Therefore, these isolates were selected for tea nursery trials. The rhizobacterial isolates were inoculated individually and also by preparing different consortia as described previously in “treatment details”. The inoculation of consortia containing all the four bacterial isolates (Treatment 11) in tea plants showed a significant (*P* < 0.05) increase in root and shoot length, fresh and dry roots and shoots weight and the number of leaves as compared to the commercial fertilizer and uninoculated control plants (Fig 3 and S2 Fig). For root and shoot length, the net length of the main roots and shoots were measured. However, the weight of the roots and shoots were measured including main and all the side roots and shoots. The PCA analysis also revealed that the microbial consortia (Treatment 11) and commercial fertilizer clustered closer to each other and the other treatments were clustered near to the uninoculated control (Fig 4).

### ARDRA, BOX-PCR fingerprinting and phylogenetic analysis

**Fig 5:**
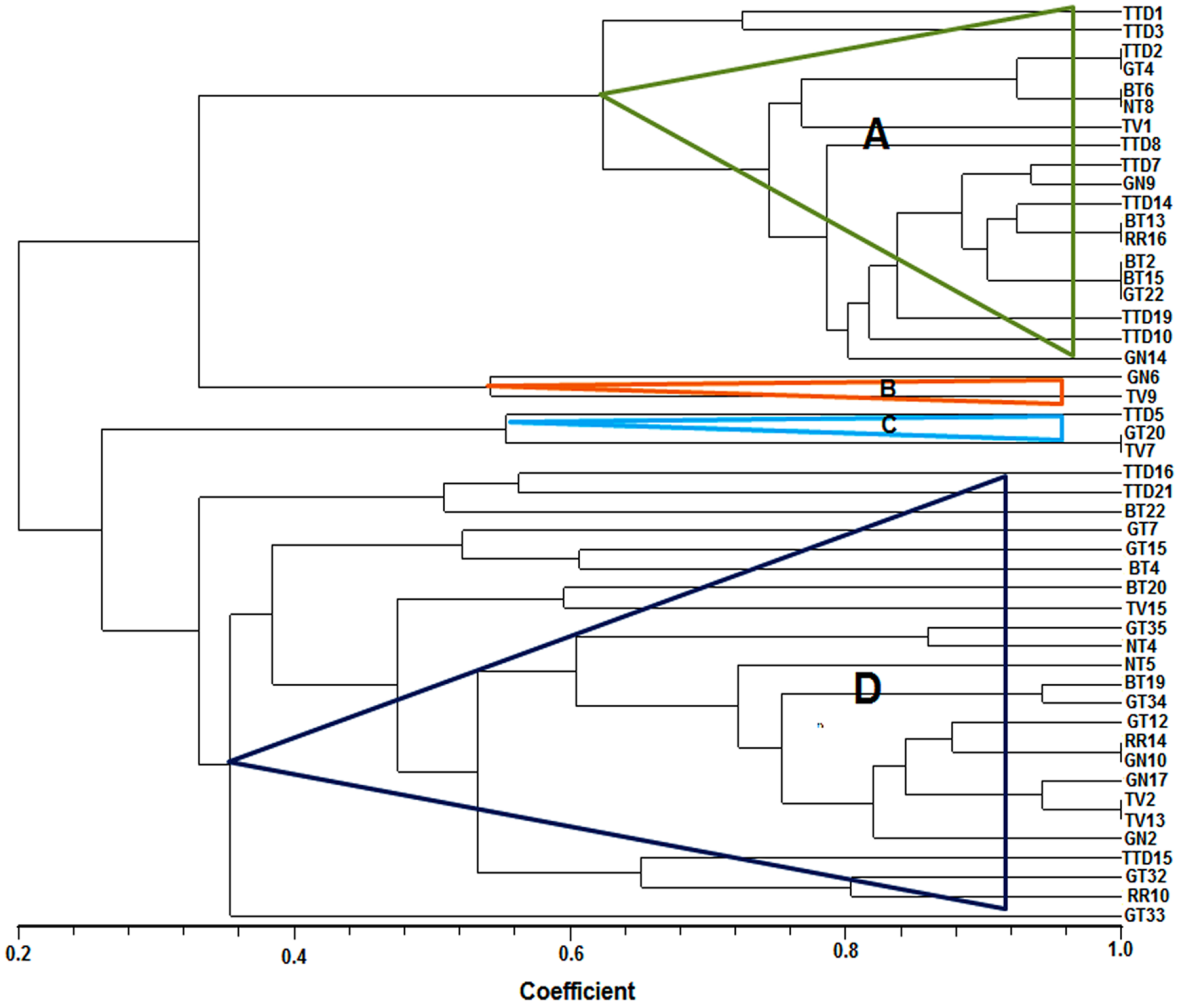
Dendrogram generated using Dice similarity coefficient index from ARDRA banding patterns of the rhizobacterial isolates using NTSYS 2.02 software. Based on the dendrogram generated the rhizobacterial isolates are divided into four major clusters A, B, C and D where B clustered with only *Pseudomonas* spp. and C clustered with only *Brevibacillus* spp.

**Fig 6:**
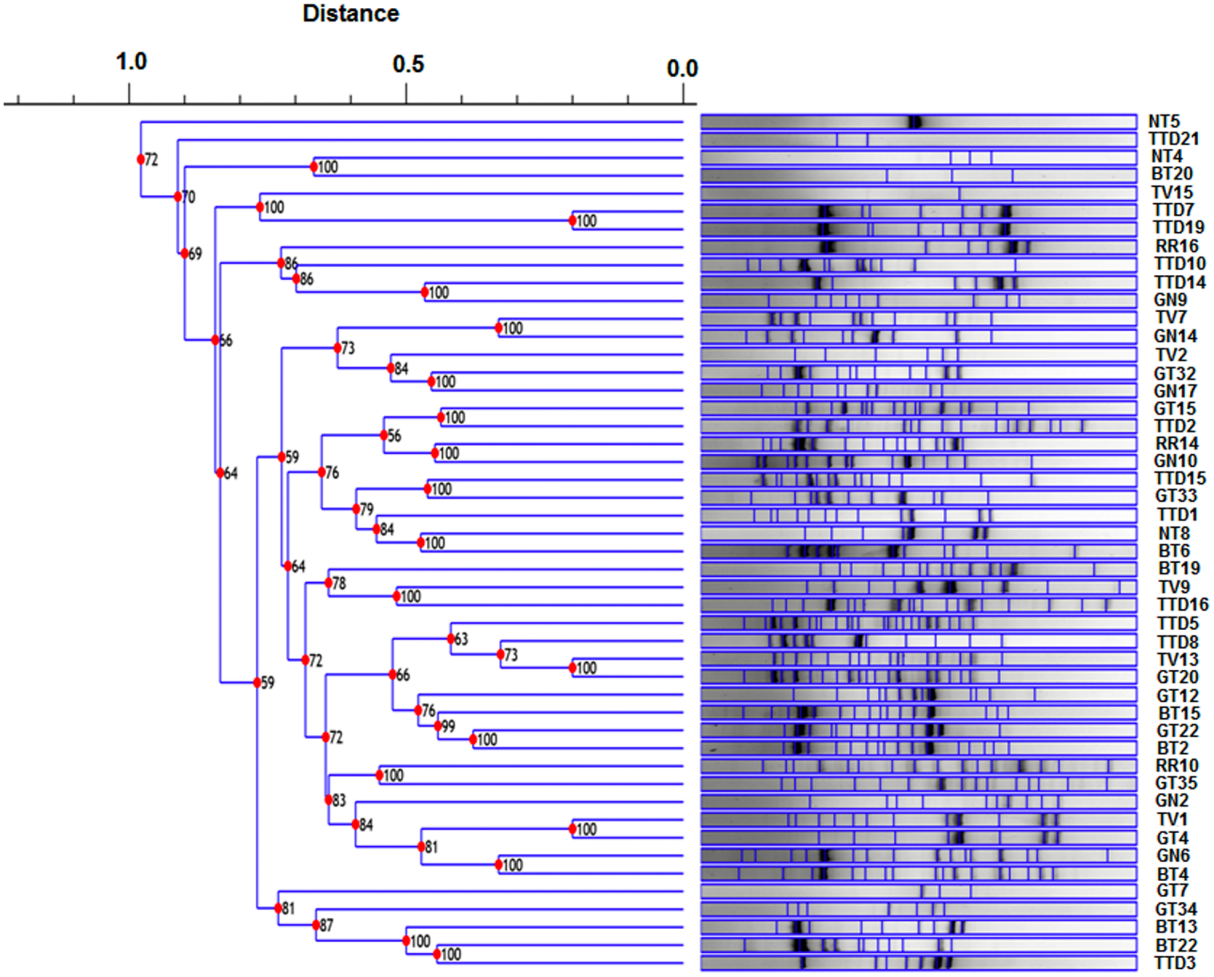
Dendrogram generated using Dice similarity coefficient index from BOX-PCR genomic fingerprints of rhizobacterial isolates using Phoretix 1D software.

**Fig 7:**
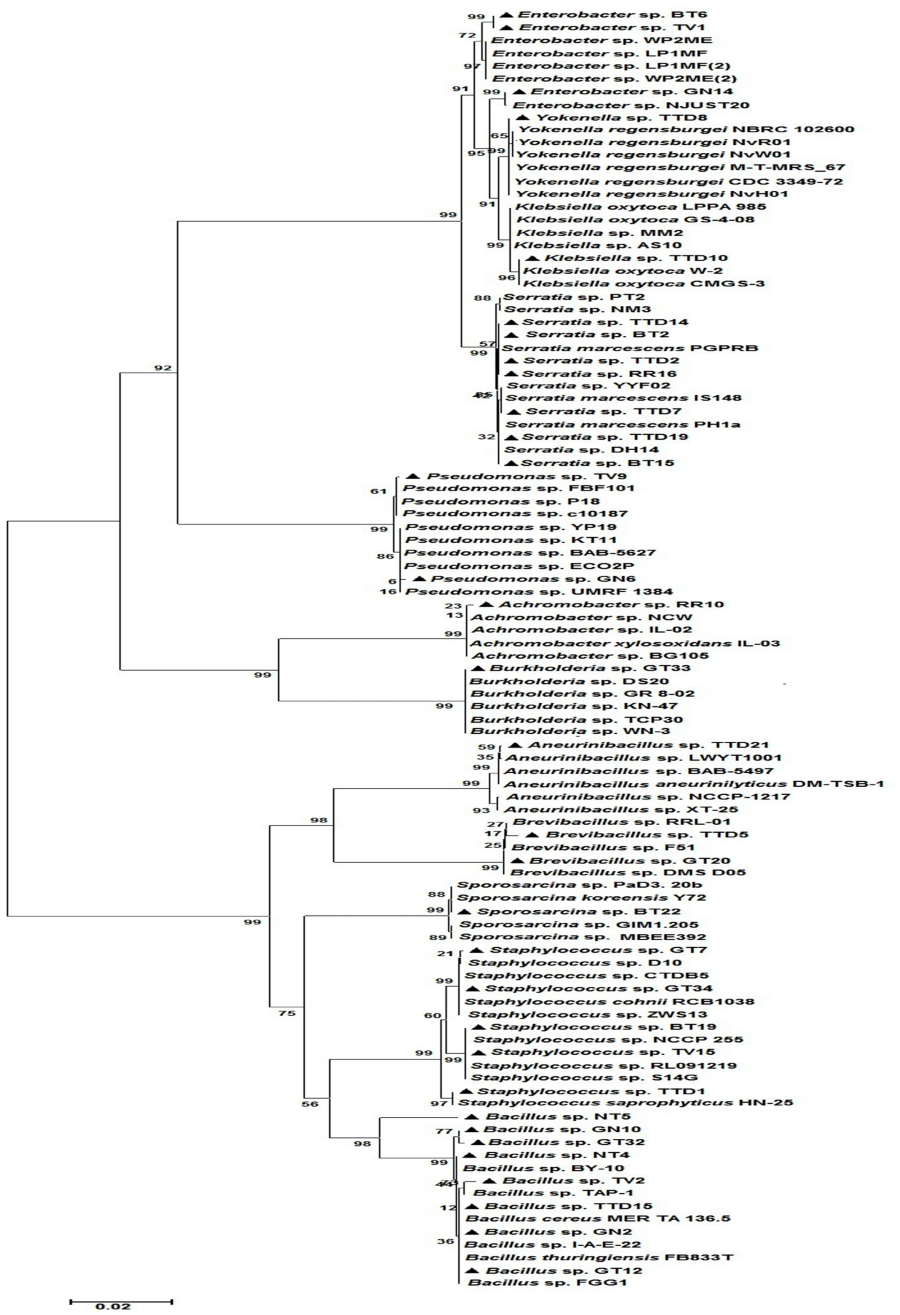
NJ-phylogenetic tree showing the evolutionary relationship between selected potential PGPR isolates and reference strains from GenBank database. The bar represents 0.05 substitutions per site, bootstrap values (*n* = 1000) are displayed.

The restriction digestion profiles of all the 48 selected antagonistic isolates were analysed and a dendrogram was constructed. The 48 isolates were divided into four major distinct clusters i.e., cluster A-D in the dendrogram (Fig 5). Among these four major clusters, B clustered with only *Pseudomonas* spp. and C clustered with only *Brevibacillus* spp. which was separated on the basis of their ARDRA banding patterns. The BOX-PCR fingerprinting of all the selected antagonistic isolates were performed and the bands of sizes between 500 bp to 5 kb were considered for scoring. The BOX-PCR generated a distinct variation in the banding pattern of the isolates indicating the presence of different genotypes among the isolates (Fig 6). For molecular identification, one representative isolate was selected from each clade of the dendrogram generated by BOX-PCR and ARDRA profiles. An NJ-dendrogram was generated using the sequences from the selected 33 rhizobacterial isolates and the representative sequences from the databases (Fig 7 and Table 4).

**Table 4:** Molecular identification of 16S rRNA gene of potent rhizobacteria, sequence accession numbers and their origin.

Sl No	Strain Code	GenBank accession no.	Base pair length	Closest sequence similarity (%)	Origin
1	TTD1	KX373959	1405	*Staphylococcus saprophyticus* (100%)	North-Tukvar tea estate
2	TTD2	KX373960	1406	*Serratia marcescens* (99.7%)	North-Tukvar tea estate
3	TTD5	KX373961	1376	*Brevibacillus agri* (99.2%)	North-Tukvar tea estate
4	TTD7	KX373962	1330	*Serratia marcescens* (99.9%)	North-Tukvar tea estate
5	TTD8	KX373963	1405	*Yokenella regensburgei* (99.5%)	North-Tukvar tea estate
6	TTD10	KX373964	1391	*Klebsiella michiganensis* (99.7%)	North-Tukvar tea estate
7	TTD14	KX373965	1409	*Serratia marcescens* (99.7%)	North-Tukvar tea estate
8	TTD15	KX373966	1402	*Bacillus cereus* (100%)	North-Tukvar tea estate
9	TTD19	KX373967	1330	*Serratia marcescens* (100%)	North-Tukvar tea estate
10	TTD21	KX373968	1401	*Aneurinibacillus aneurinilyticus* (99.8)	North-Tukvar tea estate
11	BT2	KX373969	1409	*Serratia marcescens* (99.7%)	Barnesbeg tea estate
12	BT6	KX373970	1403	*Enterobacter* sp. (99.4%)	Barnesbeg tea estate
13	BT15	KX373971	1408	*Serratia marcescens* (99.9%)	Barnesbeg tea estate
14	BT19	KX373972	1397	*Staphylococcus equorum* (100%)	Barnesbeg tea estate
15	BT22	KX373973	1398	*Sporosarcina koreensis* (99.9%)	Barnesbeg tea estate
16	GN2	KX373974	1416	*Bacillus toyonensis* (99.9%)	Ging tea estate
17	GN6	KX373975	1377	*Pseudomonas monteilii* (99.7%)	Ging tea estate
18	GN10	KX373976	1396	*Bacillus cereus* (99%)	Ging tea estate
19	GN14	KX373977	1389	*Enterobacter aerogenes* (99.6%)	Ging tea estate
20	GT7	KX373978	1402	*Staphylococcus cohnii* (99.9%)	Gielle tea estate
21	GT12	KX373979	1416	*Bacillus toyonensis* (99.9%)	Gielle tea estate
22	GT20	KX373980	1394	*Brevibacillus agri* (99.5%)	Gielle tea estate
23	GT32	KX373981	1402	*Bacillus cereus* (99%)	Gielle tea estate
24	GT33	KX373982	1382	*Burkholderia territorii* (100%)	Gielle tea estate
25	GT34	KX373983	1418	*Staphylococcus cohnii* (100%)	Gielle tea estate
26	NT4	KX373984	1404	*Bacillus cereus* (100%)	Namringtea estate
27	NT5	KX373985	1416	*Bacillus megaterium* (100%)	Namringtea estate
28	RR10	KX373986	1386	*Achromobacter xylosoxidans* (99.7%)	Rangli-Rangliot tea estate
29	RR16	KX373987	1398	*Serratia marcescens* (99.7%)	Rangli-Rangliot tea estate
30	TV1	KX373988	1405	*Enterobacter cancerogenus* (99.4%)	Teesta Valley tea estate
31	TV2	KX373989	1370	*Bacillus thuringiensis* (99.4%)	Teesta Valley tea estate
32	TV9	KX373990	1361	*Pseudomonas hunanensis* (99.8%)	Teesta Valley tea estate
33	TV15	KX373991	1414	*Staphylococcus equorum* (99.8%)	Teesta Valley tea estate

## Discussion

The Darjeeling hill area is situated in the foothills of the Himalayas with a geographical topology which provides the Darjeeling tea its unique identity. The climatic condition of the region also contributed to the inimitable flavour and aroma of Darjeeling tea. However, the geographical location and the climatic condition of Darjeeling are also congenial to different fungal infestations in tea plants. In addition, the regular use of chemicals as fertilizers, fungicides and pesticides in tea growing areas has indirectly had a negative impact on the environment [5]. Therefore, there is an immediate need to reduce the use of chemical inputs for a sustainable approach to tea cultivation. The commercial potential of PGPR application has been demonstrated in different agricultural crops and due to which some of the microbial-based bio-formulations have been commercialized [49]. After the literature review, it has been found that very little data on tea crop associated PGPR are available, especially in Northeast India.

In our study, 48 promising rhizobacterial isolates were selected out of total 150 isolates on the basis of *in vitro* antifungal and PGP assays. These isolates have exhibited multifarious antifungal traits like siderophore production, production of different hydrolytic enzymes such as chitinase, protease and cellulose which help in fungal cell wall degradation. In addition, these isolates also produced different PGP traits like IAA, ammonia, ACC deaminase and phosphate solubilization. The genetic diversity among the 48 potential PGPR isolates was studied and documented by using ARDRA and BOX-PCR genotyping analysis. Analysis of culturable bacterial diversity by these molecular tools is very suitable and convenient for species-specific fingerprint and phylogenetic analysis [38]. However, this result gives partial information concerning the culturable bacterial composition in the tea rhizosphere soil. The culture dependent method has some limitations such as many species fail to grow on the plate surface at all of a given medium and may be the dominant species on the plate surface are less which underestimate the richness of the sample [50]. Though the culturable techniques have some limitations to estimate the microbial population in environmental samples, however, it has the potential to demonstrate to retrieve both abundant and rare taxa in different environmental samples [51]. Thirty three representative isolates were selected from each group of the dendrogram generated by BOX-PCR genotyping and ARDRA profile, and they were identified by 16S rRNA gene sequencing. The BLAST similarity searches and phylogenetic analysis revealed that these 33 isolates belonged to 12 different genera. Out of these 33 isolates, 8 (24.2%) isolates belonged to genus *Bacillus*, 7 (21.2%) were from genus *Serratia*, 5 (15.2%) from genus *Staphylococcus*, 3 (9.1%) from genus *Enterobacter*, 2 (6.1%) each from genera *Brevibacillus* and *Pseudomonas*, and 1 (3.0%) isolate each belonged to genera *Burkholderia*, *Achromobacter*, *Klebsiella*, *Yokenella*, *Aneurinibacillus* and *Sporosarcina*. ARDRA profile divided these isolates into four major clusters A, B, C and D where B clustered with only *Pseudomonas* spp. and C clustered with only *Brevibacillus* spp. The Shannon-Wiener diversity index and evenness index based on the number of isolates belonging to each group of BOX-PCR and ARDRA profiles was found H = 2.17 and E = 0.88 respectively. Genetic diversity of our study showed that the genus *Bacillus* is the most abundant in tea rhizosphere soil. *Bacillus* and *Bacillus*-derived genera were also previously reported in different crops as dominant genera of rhizosphere soil. In 2008, Beneduzi et al. [17] reported *Bacillus* genus as the most abundant group among the PGPR isolated from rice. Similarly, in 2009, Upadhyay et al. [15] documented that the *Bacillus* genus was dominant in root-adhering soil of wheat under the saline condition. Jin et al. [21] and Fan et al. [52] also reported that *Bacillus* is the major genus associated with the rhizosphere soil of tobacco and *Panax notoginseng* plants.

The PGPR isolates were assessed by bonitur assessment scale and were also ranked on the basis of their *in vitro* PGP and antifungal assay as shown in Table 3. Fifteen isolates showed the most promising *in vitro* activity among the 48 PGPR isolates. However, the 16S rRNA gene sequencing of these isolates showed that most of the isolates belonged to *Enterobacter aerogenes*, *Serratia marcescens*, *Staphylococcus cohnii* and *Bacillus cereus*. From the BLAST and Ez-Taxon results, the 1st ranked isolate of assessment table, GN14 was 99.6% similar to *E*. *aerogenes*, the 2nd ranked isolate TTD7 was 99.9% similar to *S*. *marcescens*, 3rd ranked isolate TV1 was 99.4% similar to *E*. *cancerogenus*. These isolates were reported as promising PGPR in different crops including the tea plants. In 2016, Singh and Jha [53] reported that *S*. *marcescens* CDP-13 showed multifarious PGP activity and induced systemic resistance and enhanced salinity tolerance in wheat (*Triticum aestivum* L.). In 2013, Chakraborty et al. [5] reported that *S*. *marcescens* TRS-1 showed promising *in vitro* and antifungal activity and growth in the tea plants in field condition. George et al. [54] documented that *S*. *marcescens* KiSII and *Enterobacter* sp. RNF 267, which were isolated from the rhizosphere of coconut palms (*Cocos nucifera* L.), possessed diverse PGP traits. *E*. *cancerogenus* MSA2 was reported as PGPR, which stimulated the growth of *Jatropha curcas* [55]. *E*. *aerogenes* strain R43 isolated from Brazil Pine (*Araucaria angustifolia*) tree also showed very promising PGP activity [56]. Though these isolates were reported as a PGPR in different field crops, they were considered as human opportunistic pathogens. There were several reports on the pathogenicity of these isolates. *E*. *aerogenes* was isolated from respiratory, urinary, blood and gastrointestinal tract as human clinical specimens [57]. *S*. *marcescens* was also reported as a common cause of urinary tract and ocular lens infections [58]. Similarly, osteomyelitis caused by *E*. *cancerogenus* infection was also reported [59]. Therefore, tea plant growth promotion experiments in nursery conditions, the four isolates *B*. *agri* TTD5, *A*. *Aneurinilyticus* TTD21, *S*. *koreensis* BT22 and *B*. *megaterium* NT5 were selected. Review of the relevant literature showed no reports about these isolates as human opportunistic pathogens and these might therefore be considered as non-pathogenic isolates.

The selected isolates also showed multifarious *in vitro* PGP activity and enhanced the tea plant growth in nursery conditions. Among the four selected PGPR isolates, the isolate *B*. *agri* TTD5 exhibited *in vitro* chitinolytic activity and also showed the presence of glycoside hydrolase family 18 group A bacterial chitinase gene which proved the involvement of chitinolytic metabolites in this antifungal activity. Moreover, these isolates produced cellulase and protease as extracellular lytic enzymes that can degrade several components present in the cell walls of fungi and oomycetes [60]. Consequently, the isolate *B*. *agri* TTD5 showed very promising *in vitro* antifungal activity against *N*. *sphaerica*, *P*. *theae*, *G*. *cingulata*, *R*. *solani* and *F*. *oxysporum*. This may be due to the production of this wide array of extracellular enzymes. The isolates also produced different PGP traits viz., IAA, phosphate solubilization, ACC deaminase production, ammonia production and siderophore production. IAA is a vital phytohormone that plays an important role in plant growth and development. Rhizobacteria with IAA production property helps in increasing the root length and surface area, and also loosens plant cell walls, which facilitates better access to soil nutrients and establishes intense plant-microbe interaction by accumulating more root exudates [61]. Phosphate solubilization is another important mechanism of plant growth promotion by root-associated bacteria; in which rhizobacteria solubilize phosphate by producing low molecular weight organic acids which result in the acidification of the soil or media [62–64]. In our study PGPR isolates showed solubilization of tricalcium phosphate in the media with a significant decrease in pH. Ammonia production also plays an important role in plant growth by the accumulation of nitrogen and helps in promoting root and shoot growth and biomass production [65]. *A*. *aneurinilyticus* TD21 showed higher ammonia production i.e. 6 μmol mL^-1^ among the four selected PGPR. ACC deaminase is a very important enzyme related to stress relief and able to alleviate different types of biotic and abiotic stress such as effects of phytopathogenic microorganisms, stress from different heavy metals, radiation, polyaromatic hydrocarbons, high salt concentration, draught, temperature stress and flooding [66, 67]. Siderophore production is one of the important traits exhibited by PGPR. Siderophore produced by the PGPR bind the Fe^3+^ in the rhizosphere and efficiently prevent the propagation of fungal pathogens by depriving them of available iron [68, 69]. The PGPR isolates in our study showed promising siderophore production, which implies that these isolates were also capable of preventing the growth of deleterious bacteria in rhizosphere soil.

Among the isolates selected for nursery experiment, *B*. *megaterium* was previously reported as PGPR isolated from tea rhizosphere soil by Chakraborty et al. [4] and Chakraborty et al. [19] for the tea growth promotion in field condition. Porcel et al. [70] reported that *B*. *megaterium* enhanced tomato plant growth. Similarly, *B*. *brevis* strain of the genus *Brevibacillus* was reported as potential PGPR for *Eucalyptus globules* [71] and cotton (*Gossypium hirsutum*) crop [72]. Though the genus *Brevibacillus* was reported as PGPR in different crops, *B*. *agri* has not been reported as PGPR yet. After reviewing of literature it has been found that strains of *A*. *aneurinilyticus* and *S*. *koreensis* were also not reported as PGPR. Hence, to the best of our knowledge, the selected PGPR isolates obtained in the present study are documented for the first time as PGPR.

We have previously reported that the inoculation of *Enterobacter lignolyticus* strain TG1 isolated from Assam tea rhizosphere soil showed plant growth promotion in three tea clones TV1, TV19 and TV20 in greenhouse condition [20]. Chakraborty et al. (2013) also reported that the inoculation of three potent PGPR strains enhanced plant growth in five tea varieties viz., TV-18, TV-23, TV-25, TV-26 and T-17 [5]. In the present study, we have evaluated the four selected bacterial inoculums and its various consortia in two different tea clones TV1 and Teenali-17 in nursery condition. The tea plant growth was evaluated by considering the plant growth parameters such as shoot and root length, fresh and dry shoots and roots weight and the number of leaves. The multivariate PCA analysis and ANOVA analysis were performed considering these growth parameters against all treatments in two different clones. These two analyses revealed that the plants inoculated with the commercial fertilizer and consortia of all the four PGPR isolates (treatment 11) showed significantly higher growth than the uninoculated control and other treatments in both the tea clones. The growth promoting experiment was performed in commercial nursery conditions using non-sterile soil. Tea growing soil microbial community is complex and varies in composition, which represents a real challenge in soil ecology. The application of bacterial inoculums consists of high densities of viable and efficient microbes for rapid colonization of the rhizosphere soil. However, any changes in the native microbial community structure due to the inoculation could be neutralized by ecosystem resilience which is driven by plant-soil-biota [73]. Since the bacterial inoculums treatment has shown the significant increase in different growth parameters of the tea plants. Therefore, the bacterial inoculums utilized in this nursery experiment were assumed to be shown synergistic PGP effect of the selected isolates, root colonization and competent with the other native soil microflora in the rhizosphere soil of the treated tea clones.

## Conclusion

In this study, the bacteria isolated from the tea rhizosphere soil of Darjeeling have shown multifarious PGP traits and antifungal activity. The genotyping study of the tea associated PGPRs revealed the culturable bacterial community present in the tea rhizosphere soil of the Darjeeling tea estates in West Bengal, India. Exploration of region-specific tea associated microorganisms having PGP and biocontrol potential is important to obtain suitable formulations helpful in integrated nutrient management (INM) and integrated disease management (IDM) strategies. Thus, in the present study the efficient PGPR isolates evaluated in nursery conditions have commercial potential for development of microbial-based bio-formulation and application of these biofertilizers may result in a substantial decrease in the use of chemical fertilizer for the tea growing areas. However, different multi-locational field trials and the interaction of these PGPRs with other native soil microflora have to be evaluated in future to establish these PGPRs.

## Supporting information

S1 Fig PCR amplification of glycoside hydrolase family 18 group A bacterial chitinase. (M-100bp Ladder, 1–14 gene amplicons of size around 400 bp of 14 chitinase positive samples).
(TIF)

S2 Fig Evaluation of different treatments of PGPR in field experiment at Kopati tea estate in TV-1 and Teenali-17 tea clones. (A) and (B) 6 months old tea plants with PGPR inoculation. (C) and (D) Tea plants after 6 months of PGPR inoculation. (E) Showing shoot and root length and the number of the leaves of control, commercial fertilizer and the treatment 11 harvested tea plants after 6 months of treatment.
(TIF)

S1 Table Antagonistic activity of rhizobacteria against fungal phytopathogens. (PDF)

## References

[1] Majumder AB, Bera B, Rajan A. Tea Statistics: Global Scenario. Inc J Tea Sci. 2010; 8:121–124.

[2] Baby UI. An overview of blister blight disease of tea and its control. J Plantation Crops. 2002; 30:1–12.

[3] Goldman GH, Hayes C, Harman GE. Molecular and cellular biology of biocontrol *Trichoderma* spp. Trends Biotech. 1994; 12:478–482. 10.1016/0167-7799(94)90055-8 7765647

[4] Chakraborty U, Chakraborty BN, Basnet M. Plant growth promotion and induction of resistance in *Camellia sinensis* by *Bacillus megaterium*. J Basic Microbiol. 2006; 46: 186–195. doi: 10.1002/jobm.200510050 16721878 10.1002/jobm.200510050

[5] Chakraborty U, Chakraborty BN, Chakraborty AP, Sunar K, Dey PL. Plant growth promoting rhizobacteria mediated improvement of health status of tea plants. Indian J Biotechnol. 2013; 12:20–31.

[6] Calvo P, Nelson LM, Kloepper JW. Agricultural uses of plant biostimulants. Plant Soil. 2014; 383:3–41.

[7] Vejan P, Abdullah R, Khadiran T, Ismail S, Boyce AN. Role of Plant Growth Promoting Rhizobacteria in Agricultural Sustainability-A Review. Molecules. 2016; 21:573 doi: 10.3390/molecules21050573 27136521 10.3390/molecules21050573 PMC6273255

[8] Bharathi R, Vivekananthan R, Harish S, Ramanathan A, Samiyappan R. Rhizobacteria-based bio-formulations for the management of fruit rot infection in chillies. Crop Prot. 2004; 23:835–843.

[9] Siddiqui ZA, Baghel G, Akhtar MS. Biocontrol of *Meloidogyne javanica* by Rhizobium and plant growth-promoting rhizobacteria on lentil. World J Microbiol Biotechnol. 2007; 23:435–441.

[10] Ahmadzadeh M, Tehrani AS. Evaluation of fluorescent pseudomonads for plant growth promotion, antifungal activity against *Rhizoctonia solani* on common bean, and biocontrol potential. Biol Control. 2009; 48:101–107.

[11] Chithrashree, Udayashankar AC, Nayaka SC, Reddy MS, Srinivas C. Plant growth-promoting rhizobacteria mediate induced systemic resistance in rice against bacterial leaf blight caused by *Xanthomonas oryzae* pv. Oryzae. Biol Control. 2011; 59:114–122.

[12] Hammami I, Hsouna AB, Hamdi N, Gdoura R, Triki MA. Isolation and characterization of rhizosphere bacteria for the biocontrol of the damping-off disease of tomatoes in Tunisia. C. R. Biologies. 2013; 336:557–564. doi: 10.1016/j.crvi.2013.10.006 24296079 10.1016/j.crvi.2013.10.006

[13] Xu S, Kim BS. Evaluation of *Paenibacillus polymyxa* strain SC09-21 for biocontrol of Phytophthora blight and growth stimulation in pepper plants. Trop. plant pathol. 2016; doi: 10.1007/s40858-016-0077-5

[14] Beneduzi A, Peres D, Beschoren da Costa P, Zanettini MHB, Passaglia LMP. Genetic and phenotypic diversity of plant-growth promoting bacilli isolated from wheat fields in southern Brazil. Res in Microbiol. 2008; 159:244–250. 18490146 10.1016/j.resmic.2008.03.003

[15] Upadhyay SK, Singh DP, Saikia R. Genetic diversity of plant growth promoting rhizobacteria isolated from rhizospheric soil of wheat under saline condition. Curr Microbiol. 2009; 59:489–496. doi: 10.1007/s00284-009-9464-1 19701667 10.1007/s00284-009-9464-1

[16] Remans R, Beebe S, Blair M, Manrique G, Tovar E, Rao I et al Physiological and genetic analysis of root responsiveness to auxin-producing plant growth-promoting bacteria in common bean (*Phaseolus vulgaris* L.). Plant Soil. 2008; 302:149–161.

[17] Beneduzi A, Peres D, Vargas LK, Bodanese-Zanettini MH, Passaglia LMP. Evaluation of genetic diversity and plant growth promoting activities of nitrogen-fixing bacilli isolated from rice fields in South Brazil. Appl Soil Ecol. 2008; 39:311–320.

[18] Cordero P, Cavigliasso A, Príncipe A, Godino A, Jofré E, Mori G, Fischer S. Genetic diversity and antifungal activity of native Pseudomonas isolated from maize plants grown in a central region of Argentina. Sys Appl Microbiol. 2012; 35:342–351. 10.1016/j.syapm.2012.04.005 22748594

[19] Chakraborty U, Chakraborty BN, Chakraborty AP. Induction of plant growth promotion in *Camellia sinensis* by *Bacillus megaterium* and its bioformulations. World J Agri Sci. 2012; 8:104–112.

[20] Dutta J, Handique PJ, Thakur D. Assessment of culturable tea rhizobacteria isolated from tea estates of Assam, India for growth promotion in commercial tea cultivars. Front Microbiol. 2015; 6:1252 doi: 10.3389/fmicb.2015.01252 26617590 10.3389/fmicb.2015.01252 PMC4639606

[21] Jin F, Ding Y, Ding W, Reddy MS, Dilantha Fernando WG, Du B. Genetic diversity and phylogeny of antagonistic bacteria against *Phytophthora nicotianae* isolated from tobacco rhizosphere. Int J Mol Sci. 2011; 12:3055–3071. doi: 10.3390/ijms12053055 21686169 10.3390/ijms12053055 PMC3116175

[22] Dutta J, Gupta S, Handique PJ, Thakur D. First repot of Nigrospora leaf blight on tea caused by *Nigrospora sphaerica* in India. Plant dis. 2015; 99: 417 http://dx.doi.org/10.1094/PDIS-05-14-0545-PDN 10.1094/PDIS-05-14-0545-PDN 30699719

[23] El-Sayed WS, Akhkha A, El-Naggar MY, Elbadry M. *In vitro* antagonistic activity, plant growth promoting traits and phylogenetic affiliation of rhizobacteria associated with wild plants grown in arid soil. Front Microbiol. 2014; 5: 651 doi: 10.3389/fmicb.2014.00651 25538687 10.3389/fmicb.2014.00651 PMC4255609

[24] Schwyn B, Neilands JB. Universal chemical assay for the detection and determination of siderophores. Anal Biochem. 1987; 160:47–56. 2952030 10.1016/0003-2697(87)90612-9

[25] Patel AK, Deshattiwar MK, Chaudhari BL, Chincholkar SB. Production, purification and chemical characterization of the catecholate siderophore from potent probiotic strains of *Bacillus* spp. Bioresour Technol. 2009; 100:368–373. doi: 10.1016/j.biortech.2008.05.008 18585911 10.1016/j.biortech.2008.05.008

[26] Roberts WK, Selitrennikoff CP. Plant and bacterial chitinases differ in antifungal activity. J Gen Microbiol. 1988; 134:169–176.

[27] Kasana RC, Salwan R, Dhar H, Dutt S, Gulati A. A rapid and easy method for the detection of microbial cellulases on agar plates using gram’s iodine. Curr Microbiol. 2008; 57:503–507. doi: 10.1007/s00284-008-9276-8 18810533 10.1007/s00284-008-9276-8

[28] Chu WH. Optimization of extracellular alkaline protease production from species of *Bacillus*. J Ind Microbiol Biotechnol. 2007; 34:241–245. doi: 10.1007/s10295-006-0192-2 17171551 10.1007/s10295-006-0192-2

[29] Gordon SA, Weber RP. Colorimetric estimation of indole-acetic acid. Plant Physiol. 1951; 26:192–195. 16654351 10.1104/pp.26.1.192 PMC437633

[30] Katznelson H, Bose B. Metabolic activity and phosphate-dissolving capability of bacterial isolates from wheat roots, rhizosphere, and non-rhizosphere soil. Can. J. Microbiol. 1959; 5:79–85. 13629388 10.1139/m59-010

[31] Cappuccino JC, Sherman N. Negative staining In: Cappuccino JC, Sherman N, editors. Microbiology: A Laboratory Manual. Benjamin/Cummings Redwood City; 1992 pp. 125–179.

[32] Dworkin M, Foster J. Experiments with some microorganisms which utilize ethane and hydrogen. J Bacteriol. 1958; 75:592–601. 13538930 10.1128/jb.75.5.592-603.1958 PMC290115

[33] Penrose DM, Glick BR. Methods for isolating and characterizing ACC deaminase-containing plant growth-promoting rhizobacteria. Physiol Plant. 2003; 118:10–15. 12702008 10.1034/j.1399-3054.2003.00086.x

[34] Ryu RJ, Patten CL. Aromatic amino acid-dependent expression of indole-3-pyruvate decarboxylase is regulated by TyrR in Enterobacter cloacae UW5. J Bacteriol. 2008; 190:7200–7208. doi: 10.1128/JB.00804-08 18757531 10.1128/JB.00804-08 PMC2580706

[35] Fiske CH, Subbarow Y. A colorimetric determination of phosphorus. J Biol Chem. 1925; 66:375–400.

[36] Goswami D, Dhandhukia P, Patel P, Thakker JN. Screening of PGPR from saline desert of Kutch: Growth promotion in *Arachis hypogea* by *Bacillus licheniformis* A2. Microbiol Res. 2014; 169: 66–75. doi: 10.1016/j.micres.2013.07.004 23896166 10.1016/j.micres.2013.07.004

[37] Weisburg WG, Barns SM, Lane DJ. 16S ribosomal DNA amplification for phylogenetic study. J Bacteriol. 1991; 173:697–703. 1987160 10.1128/jb.173.2.697-703.1991 PMC207061

[38] Mishra RK, Pandey BK, Pathak N, Zeeshan M. BOX-PCR- and ERIC-PCR-based genotyping and phylogenetic correlation among *Fusarium oxysporum* isolates associated with wilt disease in *Psidium guajava* L. Biocatal Agri Biotechnol. 2015; 4:25–32.

[39] Chun J, Lee JH, Jung Y, Kim M, Kim S, Kim BK, et al EzTaxon: a web-based tool for the identification of prokaryotes based on 16S ribosomal RNA gene sequences. Int J Syst Evol Microbiol. 2007; 57:2259–2261. doi: 10.1099/ijs.0.64915-0 17911292 10.1099/ijs.0.64915-0

[40] Tamura K, Peterson D, Peterson N, Stecher G, Nei M, Kumar S. MEGA5: molecular evolutionary genetics analysis using maximum likelihood, evolutionary distance, and maximum parsimony methods. Mol Biol Evol. 2011; 28:2731–2739. doi: 10.1093/molbev/msr121 21546353 10.1093/molbev/msr121 PMC3203626

[41] Kimura MA. Simple method for estimating evolutionary rates of base substitutions through comparative studies of nucleotide sequences. J Mol Evol. 1980; 16:111–120. 7463489 10.1007/BF01731581

[42] Felsenstein J. Confidence limits on phylogenies: an approach using the bootstrap. Evolution. 1985; 39:783–791. doi: 10.1111/j.1558-5646.1985.tb00420.x 28561359 10.1111/j.1558-5646.1985.tb00420.x

[43] Williamson N, Brian P, Wellington EMH. Molecular detection of bacterial and streptomycete chitinases in the environment. Antonie van Leeuwenhoek. 2000; 78: 315–321. 11386354 10.1023/a:1010225909148

[44] Martin B, Chadwick W, Yi T, Park SS, Lu D, Ni B, et al VENNTURE-A novel Venn diagram investigational tool for multiple pharmacological dataset analysis. Plos one. 2012; 7:e36911 doi: 10.1371/journal.pone.0036911 22606307 10.1371/journal.pone.0036911 PMC3351456

[45] Verma VC, Gond SK, Kumar A, Kharwar RN, Strobel G. The endophyticmycoflora of bark, leaf, and stem tissues of *Azadirachta indica* A. Juss (Neem) from Varanasi (India). Microb Ecol. 2007; 54:119–25. doi: 10.1007/s00248-006-9179-9 17394041 10.1007/s00248-006-9179-9

[46] Xia J, Sinelnikov I, Han B, Wishart DS. MetaboAnalyst 3.0-making metabolomics more meaningful. Nucleic Acids Res. 2015; 43: 251–257. doi: 10.1093/nar/gkv380 25897128 10.1093/nar/gkv380 PMC4489235

[47] USDA (2010). “Soil taxonomy,” A Basic System of Soil Classification for Making and Interpreting Soil Surveys. Washington, DC: United States Department of Agriculture.

[48] Passari AK, Mishra VK, Gupta VK, Yadav MK, Saikia R, Singh BP. *In vitro* and *in vivo* plant growth promoting activities and DNA fingerprinting of antagonistic endophytic actinomycetes associates with medicinal plants. PLoS one. 2015; 10(9): e0139468 doi: 10.1371/journal.pone.0139468 26422789 10.1371/journal.pone.0139468 PMC4589368

[49] Nakkeeran S, Fernando WGD, Siddiqui ZA. Plant growth promoting rhizobacteria formulations and its scope in commercialization for the management of pests and diseases In: Siddiqui ZA, editor. PGPR: biocontrol and biofertilization. Springer, Dordrecht; 2005 pp. 257–296.

[50] Kisand V, Wikner J. Combining culture-dependent and -independent methodologies for estimation of richness of estuarine bacterioplankton consuming riverine dissolved organic matter. Appl Env Microbiol. 2003; 69:3607–3616. 12788769 10.1128/AEM.69.6.3607-3616.2003 PMC161508

[51] França L, Sannino C, Turchetti B, Buzzini P, Margesin R. Seasonal and altitudinal changes of culturable bacterial and yeast diversity in Alpine forest soils. Extremophiles. 2016; 20:855–873. doi: 10.1007/s00792-016-0874-2 27620454 10.1007/s00792-016-0874-2 PMC5085987

[52] Fan ZY, Miao CP, Qiao XG, Zheng YK, Chen HH, Chen YW, et al Diversity, distribution, and antagonistic activities of rhizobacteria of *Panax notoginseng*. J Ginseng Res. 2016; 40:97–104. doi: 10.1016/j.jgr.2015.05.003 27158229 10.1016/j.jgr.2015.05.003 PMC4845043

[53] Singh RP, Jha PN. The multifarious PGPR *Serratia marcescens* CDP-13 augments induced systemic resistance and enhanced salinity tolerance of wheat (*Triticum aestivum* L.). PLos one. 2016; 11(6): e0155026 doi: 10.1371/journal.pone.0155026 27322827 10.1371/journal.pone.0155026 PMC4913913

[54] George P, Gupta A, Gopal M, Thomas L, Thomas GV. Multifarious beneficial traits and plant growth promoting potential of *Serratia marcescens* KiSII and *Enterobacter* sp. RNF 267 isolated from the rhizosphere of coconut palms (*Cocos nucifera* L.). World J Microbiol Biotechnol. 2013; 29:109–117. doi: 10.1007/s11274-012-1163-6 22948479 10.1007/s11274-012-1163-6

[55] Jha CK, Patel B, Saraf M. Stimulation of the growth of *Jatropha curcas* by the plant growth promoting bacterium *Enterobacter cancerogenus* MSA2. World J Microbiol Biotechnol. 2012; 28:891–899. doi: 10.1007/s11274-011-0886-0 22805809 10.1007/s11274-011-0886-0

[56] Ribeiro CM, Cardoso EJBN. Isolation, selection and characterization of root-associated growth promoting bacteria in Brazil Pine (*Araucaria angustifolia*). Microbiol Res. 2012; 167:69–78. doi: 10.1016/j.micres.2011.03.003 21596540 10.1016/j.micres.2011.03.003

[57] Langley JM, Hanakowski M, Leblanc JC. Unique epidemiology of nosocomial urinary tract infection in children. Am J Infect Control. 2001; 29: 94–98. doi: 10.1067/mic.2001.111537 11287876 10.1067/mic.2001.111537

[58] Hertle R. Schwarz H. *Serratia marcescens* internalization and replication in human bladder epithelial cells. BMC Infectious Diseases. 2004; 4:16 doi: 10.1186/1471-2334-4-16 15189566 10.1186/1471-2334-4-16 PMC441377

[59] Garazzino S, Aprato A, Maiello A, Masse A, Biasibetti A, De Rosa FG et al Osteomyelitis caused by *Enterobacter cancerogenus* infection following a traumatic injury: Case Report and Review of the Literature. J Clin Microbiol. 2005; 43:1459–1461. doi: 10.1128/JCM.43.3.1459-1461.2005 15750133 10.1128/JCM.43.3.1459-1461.2005 PMC1081281

[60] Chet I, Inbar J. Biological control of fungal pathogens. Appl Biochem Biotechnol. 1994; 48:37–43. 7979350 10.1007/BF02825358

[61] Glick BR. Plant growth-promoting bacteria: mechanisms and applications. Scientifica. 2012; doi: 10.6064/2012/963401 24278762 10.6064/2012/963401 PMC3820493

[62] Gyaneshwar P, Parekh LJ, Archana G, Poole PS, Collins MD, Hutson RA, et al Involvement of a phosphate starvation inducible glucose dehydrogenase in soil phosphate solubilization by *Enterobacter asburiae*. FEMS Microbiol Lett. 1999; 171: 223–229.

[63] Puente ME, Bashan Y, Li CY, Lebsky VK. Microbial populations and activities in the rhizoplane of rock-weathering desert plants. I. Root colonization and weathering of igneous rocks. Plant Biol. 2004; 6:629–642. doi: 10.1055/s-2004-821100 15375735 10.1055/s-2004-821100

[64] Khan MS, Zaidi A, Ahmad E. Mechanism of phosphate solubilization and physiological functions of phosphate-solubilizing microorganisms In: Khan MS, Zaidi A, Mussarrat J, editors. Phosphate solubilizing microorganisms. Springer International Publishing Switzerland; 2014 pp. 34–35.

[65] Marques APGC, Pires C, Moreira H, Rangel AOSS, Castro PML. Assessment of the plant growth promotion abilities of six bacterial isolates using *Zea mays* as indicator plant. Soil Biol Biochem. 2010; 42:1229–1235.

[66] Glick BR, Cheng Z, Czarny J, Duan J. Promotion of plant growth by ACC deaminase-producing soil bacteria. Eur J Plant Pathol. 2007; 119:329–339.

[67] Lugtenberg B, Kamilova F. Plant-growth-promoting rhizobacteria. Annu Rev Microbiol. 2009; 63:541–556. doi: 10.1146/annurev.micro.62.081307.162918 19575558 10.1146/annurev.micro.62.081307.162918

[68] Kloepper JW, Leong J, Teintze M, Schroth MN. Enhanced plant growth by siderophores produced by plant growth-promoting rhizobacteria. Nature. 1980; 286: 885–886.

[69] O’Sullivan DJ, O’Gara F. Traits of fluorescent *Pseudomonas* sp. involved in suppression of plant root pathogens. Microbiol. Res. 1992; 56:662–676. 10.1128/mr.56.4.662-676.1992 PMC372893 1480114

[70] Porcel R, Zamarreño ÁM, García-Mina JM, Aroca R. Involvement of plant endogenous ABA in *Bacillus megaterium* PGPR activity in tomato plants. BMC Plant Biology. 2014; 14:36–48. doi: 10.1186/1471-2229-14-36 24460926 10.1186/1471-2229-14-36 PMC3903769

[71] Dίaz K, Valiente C, Martı´nez M, Castillo M, Sanfuentes E. Root-promoting rhizobacteria in *Eucalyptus globules* cuttings. World J Microbiol Biotechnol. 2009; 25: 867–873. 10.1007/s11274-012-1003-8 22806022

[72] Nehra V, Saharan BS, Choudhary M. Evaluation of *Brevibacillus brevis* as a potential plant growth promoting rhizobacteria for cotton (*Gossypium hirsutum*) crop. Springerplus. 2016; doi: 10.1186/s40064-016-2584-8 27386392 10.1186/s40064-016-2584-8 PMC4929095

[73] Trabelsi D, Mhamdi R. Microbial inoculants and their impact on soil microbial communities: a review. BioMed Res Int. 2013; doi: 10.1155/2013/863240 23957006 10.1155/2013/863240 PMC3728534

