# Supplementary figures and images for: Evaluation of multifarious plant growth promoting traits, antagonistic potential and phylogenetic affiliation of rhizobacteria associated with commercial tea plants grown in Darjeeling, India
Source: PLoS One. 2017 Aug 3;12(8):e0182302. doi: 10.1371/journal.pone.0182302 (PMC5542436; doi:10.1371/journal.pone.0182302)

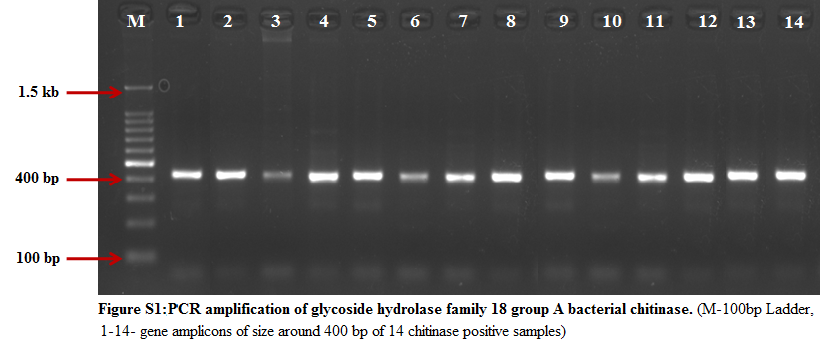

Supplement: S1 Fig — (M-100bp Ladder, 1–14 gene amplicons of size around 400 bp of 14 chitinase positive samples). (TIF) [file pone.0182302.s001.tif]

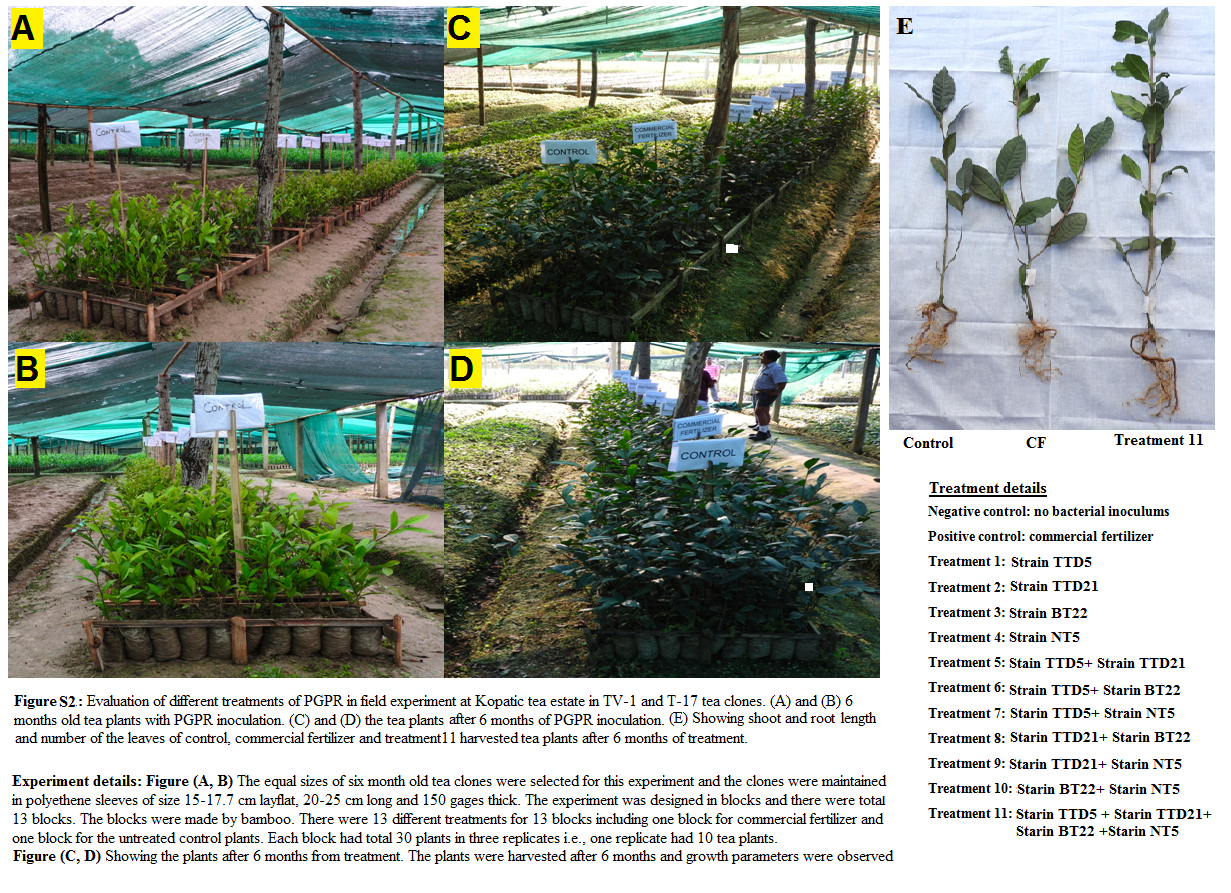

Supplement: S2 Fig — (A) and (B) 6 months old tea plants with PGPR inoculation. (C) and (D) Tea plants after 6 months of PGPR inoculation. (E) Showing shoot and root length and the number of the leaves of control, commercial fertilizer and the treatment 11 harvested tea plants after 6 months of treatment. (TIF) [file pone.0182302.s002.tif]
