# Supplementary material for: Evaluation of multifarious plant growth promoting traits, antagonistic potential and phylogenetic affiliation of rhizobacteria associated with commercial tea plants grown in Darjeeling, India
Source: PLoS One. 2017 Aug 3;12(8):e0182302. doi: 10.1371/journal.pone.0182302 (PMC5542436; doi:10.1371/journal.pone.0182302)
Supplement: S1 Table — (PDF) [file pone.0182302.s003.pdf]

S1 Table. Antagonistic activity of rhizobacteria against fungal phytopathogens.

Sl No.	Strain Code	*N. sphaerica*	*P. theae*	*C. eragrostidis*	*G. cingulata*	*R. solani*	*F. oxysporum*
		Growth inhibition (%)					
1	TTD1	8.6±0.1	-	6.6±0.1	-	25±0.1	-
2	TTD2	23.6±0.3	-	-	10.5±0.1	22.5±0.1	-
3	TTD3	-	7.8±0.2	-	15±0.1	-	20.5±0.1
4	TTD5	38.5±0.2	13.1±0.1	-	27.5±0.3	35±0.4	32.5±0.2
5	TTD7	34.2±0.3	18.4±0.2	30±0.5	35±0.3	27.5±0.3	40±0.5
6	TTD8	17.9±0.2	10.5±0.1	-	-	5±0.1	-
7	TTD10	5.1±0.2	2.6±0.1	-	-	-	27.5±0.5
8	TTD14	-	2.6±0.1	-	5±0.1	-	-
9	TTD15	23.1±0.1	5.2±0.1	26.6±0.3	28.7±0.2	-	25±0.1
10	TTD16	20.5±0.1	5.2±0.1	-	-	-	30±0.3
11	TTD19	7.7±0.2	13.2±0.1	16.6±0.2	-	10±0.1	-
12	TTD21	-	5.2±0.1	-	-	17.5±0.1	-
13	BT2	30.8±0.3	-	33.3±0.2	-	30±0.3	37.5±0.2
14	BT4	-	23.6±0.5	-	-	5±0.1	-
15	BT6	5.1±0.2	26.3±0.3	23.3±0.1	-	37.5±0.5	-
16	BT13	5.1±0.1	-	16±0.1	-	20±0.1	-
17	BT15	28.2±0.2	7.9±0.1	31.6±0.3	-	30±0.4	-
18	BT19	7.1±0.4	-	-	10±0.1	-	-
19	BT20	-	19.7±0.1	-	7.5±0.1	-	-
20	BT22	3.8±0.1	-	-	-	-	12.5±0.1
21	GN2	5.9±0.1	-	-	14.5±0.1	-	-
22	GN6	17.9±0.1	23.7±0.1	6.6±0.1	-	-	-
23	GN9	-	21.1±0.1	-	-	15±0.1	20±0.1
24	GN10	12.8±0.1	39.5±0.7	-	-	-	-
25	GN14	32.5±0.2	25.2±0.1	36.6±0.5	37±0.6	30±0.1	39±0.3
26	GN17	43.6±0.5	28.9±0.5	14.6±0.1	-	-	-
27	GT4	23.1±0.2	36.8±0.3	29.3±0.2	-	27.5±0.3	-
28	GT7	-	22.5±0.1	33.3±0.2	27.5±0.3	-	25±0.1
29	GT12	-	2.1±0.2	-	-	-	-
30	GT15	-	31.5±0.1	-	17.5±0.1	-	-
31	GT20	25.6±0.2	5.2±0.1	20±0.1	-	32.5±0.3	-
32	GT22	5.1±0.1	-	4.3±0.1	-	-	-
33	GT32	5.1±0.1	23.7±0.1	37±0.3	22.5±0.2	-	-
34	GT33	-	2.6±0.1	-	-	-	32.5±0.3
35	GT34	33.3±0.3	13.2±0.1	30±0.3	30±0.2	-	-
36	GT35	2.6±0.1	23.7±0.1	-	-	-	37.5±0.5
37	TV1	41±0.4	34.2±0.6	23.3±0.1	-	15±0.1	25±0.2
38	TV2	3.8±0.1	-	-	12.5±0.1	-	-
39	TV7	-	18.4±0.2	19.3±0.1	-	-	-
40	TV9	-	12.5±0.1	28.6±0.1	12.5±0.1	12.5±0.1	22.5±0.1
41	TV13	10.2±0.1	-	-	-	17.5±0.1	-
42	TV15	-	2.6±0.1	-	-	-	10±0.1
43	NT4	23±0.1	10.5±0.1	32.6±0.5	-	25±0.2	-
44	NT5	2±0.1	-	-	-	-	-
45	NT8	-	1.3±0.1	-	17.5±0.1	-	-
46	RR10	-	10.5±0.1	17.3±0.1	-	25±0.1	-
47	RR14	23±0.1	-	-	-	-	17.5±0.1
48	RR16	15.3±0.5	-	-	-	-	21.5±0.1
